# Transcatheter bicuspid venous valve prostheses: fluid mechanical performance testing of artificial nonwoven leaflets

**DOI:** 10.1186/s12938-024-01316-x

**Published:** 2024-11-29

**Authors:** Andreas Götz, Sabine Illner, Nicklas Fiedler, Julia Schubert, Jan Oldenburg, Heinz Müller, Wolfram Schmidt, Klaus-Peter Schmitz, Niels Grabow, Kerstin Lebahn

**Affiliations:** 1 Institute for Biomedical Engineering, Rostock University Medical Center, Friedrich-Barnewitz-Str. 4, 18119 Rostock, Germany; 2 Institute for ImplantTechnology and Biomaterials e.V., Friedrich-Barnewitz-Str. 4, 18119 Rostock, Germany; 3 CORTRONIK GmbH, Friedrich-Barnewitz-Str. 4a, 18119 Rostock, Germany

**Keywords:** CVD, CVI, Venous valve, Chronic venous disease, Valve implant, Testing, Forward flow, Reflux

## Abstract

**Background:**

Chronic venous insufficiency (CVI) is a common disease with a high prevalence. Incompetent venous valves are considered as one of the main causes. Besides compression therapy, various surgical therapies are practiced, whereby the reconstruction of valves is of central importance. There is an unmet clinical need, no valve prosthesis is commercially available to date. This work introduces two versions of a patented prosthetic bicuspid valve design made of electrospun thermoplastic silicone polycarbonate polyurethane (TSPCU) nanofiber leaflets attached in a nitinol stent, and their performance in static and pulsatile operation.

**Results:**

The valves mainly fulfill the requirements widely accepted in literature. Valves of both versions were functional in the physiological pressure range up to 50 mmHg with design specific differences.

**Conclusions:**

The here introduced design versions act as a platform technology and can be tailored for an intended implantation site. Evaluation of the original and modified valve concept demonstrated efficacy, with limitations at higher loads for original design. At the current state, the modification is preferable for fabrication, as one processing step is eliminated. Moreover, specific design recommendations could be drawn for valves of similar basic structure. Future work will focus on long-term performance and biocompatibility prior to the initiation of preclinical in vivo studies.

**Supplementary Information:**

The online version contains supplementary material available at 10.1186/s12938-024-01316-x.

## Introduction

Chronic venous insufficiency (CVI), chronic venous disease (CVD), or chronic deep venous insufficiency (DVI) are often synonymously used terms, which describe principally a vascular disorder in which venous return is compromised. CVI is a persistent, progressive and often underestimated condition that is prevalent in the general population and has a huge socio-economic, physical and psychological impact.

The clinical presentation is accompanied by severe manifestations of edema, skin changes or leg ulcers. Prevalence data of CVI vary widely from around 1–40% in women and around 1–17% in men [1]. There are two pathogeneses universally accepted, primary valvular incompetence and primary congenital vein wall weakness [2]. Furthermore, there are numerous genetic candidate loci, but still rudimentarily understood [3].

Therapeutic methods for CVI demonstrate a wide variety. The standard initial treatment is compressing therapy, supported by medication [2]. Besides, there are several surgical treatment concepts, such as interventional therapies, occlusion, or venoplasty [2, 4]. For example, the “BlueLeaf” system is a minimally invasive technique to form fully autogenous deep endovenous valves using an expandable dissection tool [5]. The recurrence of varicose veins after surgery is a frequent phenomenon, which may be caused by the so called “hemodynamic paradox” [6, 7].

Venous valve prostheses are supposed to address the shortcomings of reconstruction surgery. In general, valve prostheses consist of a support structure, which in most cases is a stent, and the actual valve, which is designed as a single or multi leaflet. The stent is usually made of nitinol and the leaflet material can consist of natural or polymeric fiber mats. New generation of biostable medical polyurethane elastomers with favorable bio- and hemocompatibility is thermoplastic silicone polycarbonate urethanes (TSPCU), which can be additionally modified [8–12]. For example, polyhedral oligomeric silsesquioxanes–polycarbonate urethane (POSS–PCU) nanocomposites showed good biocompatibility, mechanical durability, tear resistance, and better thrombosis resistance than other common polymeric valve materials such as polytetrafluoroethylene (PTFE) [13–15]. In contrast, bioresorbable, co-growing and minimally invasive venous valve concepts with degradable polymers such as polycaprolactone (PCL), polydioxanone (PDS), polylactide (PLA) and polyglycolide (PGA) are also gaining ground [10, 16]. However, the associated research effort is considerably more complex, particularly with regard to inflammation, tissue regeneration processes and degradation rates. In general, published studies regarding artificial valve materials and designs are difficult to compare due to their individuality in terms of limits or technical parameters. Various models are being developed from concept studies [17], over animal studies (e.g. “SailValve”, “BVV1”– “BVV3”) [18–20], to clinical testing (e.g., “VenoValve”, “enVVe”, and “Innovein”) [21–26]. In addition, there are currently limited data to support vein reconstruction and no consensus on the characteristics of an ideal prosthesis [27–30] and no international “gold standard” in terms of operating conditions and performance requirements for venous valve prostheses. Ideally, the implant should allow free blood flow towards the heart, but prevent flow in retrograde direction. However, this point of view is still controversial, as a leakage in the valve can promote the long-term patency of the design by preventing thrombosis [19, 20].

In the development of new venous valve prostheses, generally recognized requirements, especially physiological conditions and several design aspects have to be considered to ensure safe operation. Cardiac output at rest is about 5 L/min and may reach 20 L/min at strong exercise [31, 32]. Unilateral lower limb venous blood flow is in a range of 420–450 mL/min in relaxed condition [33, 34], 400–800 mL/min at normal walking [35], 1600 mL/min at standing ankle flexion [36], and 2000 or 2450 mL/min at exercise of 40 W [37, 38] or 65 W [34], respectively. The blood flow in the legs does not differ between age groups when cycling at low intensity (20–40 W), but is significantly attenuated in older compared to younger subjects when the exercise intensity increases [39]. The anaerobic threshold describes the highest possible continuous intensity of exertion and is specified as 30–40 W for untrained older people [40].

The venous pressure at relaxed upright position depends mostly on gravitational pressure, and may reach 100 mmHg [27]. Activation of the calf muscle pump by walking removes the blood within some steps, ideally without reflux. The constantly incoming fresh blood does not fill the complete vein within one step, this creates a balance with low average pressure of 22–24 mmHg between the blood pumped out and the blood flowing in [41]. The normal operating pressure of a valve in the thigh is estimated with approximately 50 mmHg, in case no competent valve exists proximal. During coughing, sneezing, jumping or similar actions, short-term pressure surges up to 300 mmHg are transmitted from the abdomen to the legs, but in the same way to the arterial and venous system [42–45].

The vein size differs individually in a wide range as reported by various authors. Diameters of the common femoral vein are indicated with varying values of 11–12 mm [46], 6–21 mm [47], 10 mm [36], or for the proximal femoral vein of 4–16 mm [47], 8–16 mm [48], and for the distal femoral vein of 3–13 mm [47], or 3–10 mm [48]. However, vein diameters change not only due to exertion, but also with age and throughout the day. The femoral veins dilate by a few millimeters from morning to the afternoon, as indicated by 1.3–3.3 mm [49]. Suggested implant sites are 5–10 cm proximal to the saphenofemoral junction or in V. saphena parva [48]. The mid-thigh region of the femoral vein is likewise suitable [23].

From the physiological considerations further venous valve design requirements are derived. A minimal invasive surgery type is preferred and the valve design needs to ensure a blood flow similar to the native valves [29]. The orifice area should be larger than 30% of the vessel cross-sectional area [13, 50]. Leakage and reflux are controversially discussed, the valve can be considered functional when the leakage is less than 10% of the forward blood volume [51], or less than 3 mL/min [27]. Since native vein valves typically open with a pressure gradient of less than 5 mmHg, the opening pressure of prosthetic valves should be similar [27, 35, 51]. Tilting of the valve should be prevented from a fluidic perspective to keep patency and avoid thrombosis as main complication [29, 52].

Oversizing is a suitable method for secure fitting the implant in the vein, size mismatch of protheses and vein should be less than 15% [53], which means stent oversizing in the range of 1–2 mm. A rarely specified but crucial factor is which condition was used as the basis to calculate oversizing for determining the implant diameter to take account of the varying vein diameters under different load situations. Further anti-displacement techniques such as barbs are a common and effective solution to prevent valve migration, in sheep model barb penetration through the vein wall was seen in 56% of cases [54].

Recently, our group has reported on a novel prosthetic venous valve concept [55]. Main objective of the current study is to further improve this concept regarding the prevention of tilting and downstream migration, as well as maximization of effective orifice area (EOA). As part of this approach, two valve design embodiments were systematically assessed in vitro in an effort to establish more robust design and testing requirements for prosthetic venous valves. For the oversizing values, the inner diameters of the test attachments were applied as base value and set in relation to the uncompressed stent diameters. Systematic experimental testing is conducted to identify design strengths and weaknesses. The evaluation and comparison of valve performance is used to demonstrate the impact of design changes, to identify key elements for future implant developments.

## Results

### Artificial bicuspid venous valves

**Fig. 1:**
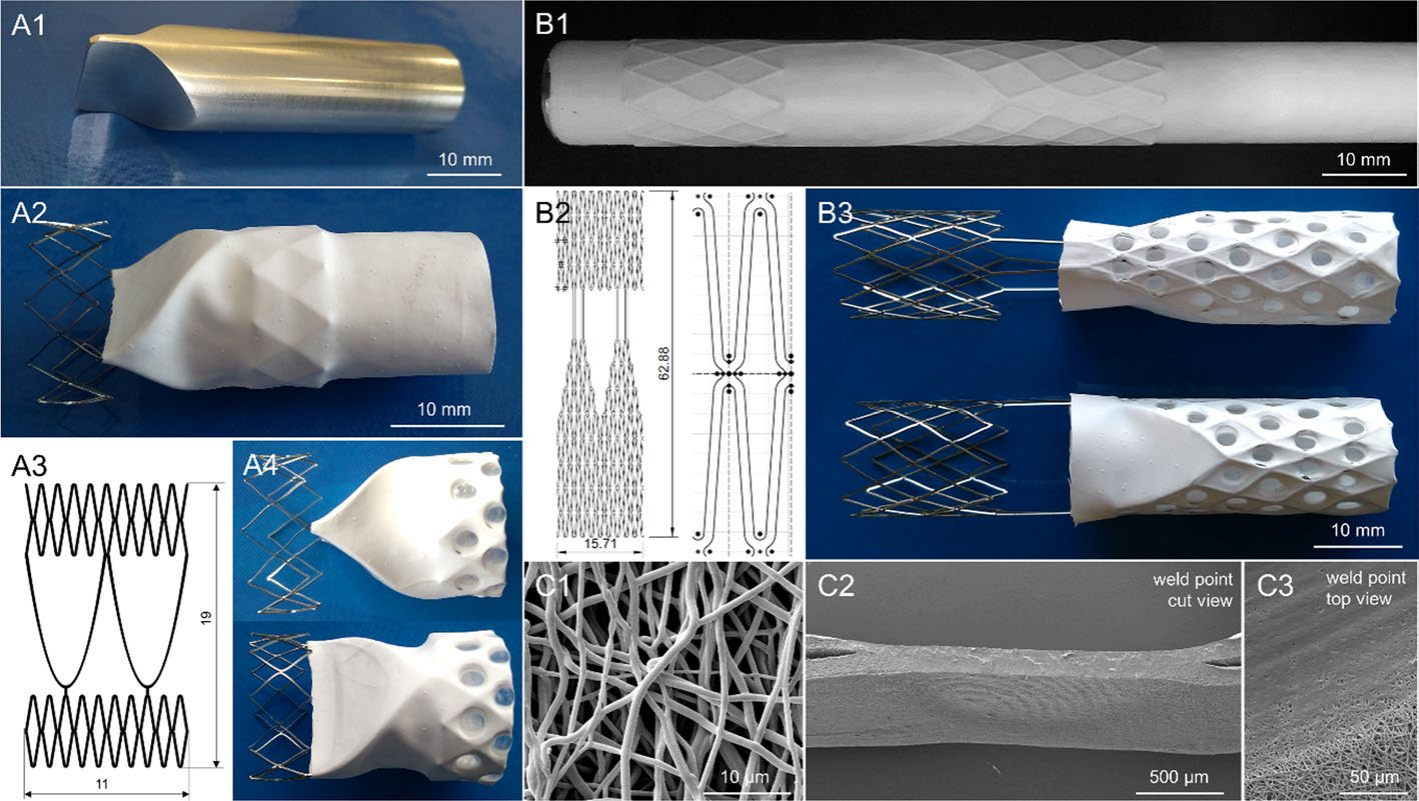
Valve versions. **A1**: aluminium mold for electrospinning of version A nonwovens, **A2**: version A, stent enclosed with electrospun cover and protruding part for fixation, **A3**: sketch of version A stent, **A4**: assembled valve of version A in front and side view, **B1**: electrospun layer on version B stent mounted on metal cylinder, **B2**: sketch of version B stent as whole and a basic element, **B3**: assembled valve of version B in front and side view, **C1**: SEM image of nanofiber material, **C2**: SEM image of a cut weld point in side view, **C3**: SEM image of a weld point in top view

In both designs, the electrospun nanofiber nonwovens were assembled in the stents as an inwardly folded, double-layered sandwich. Both sandwich layers, inside and outside of the stent structure, are thermally spot welded in the center of the rhomboid stent cells allowing clearance and load distribution, thus enabling structural flexibility during the entire load history and avoiding critical load peaks. In the welding points fibers were melted together and no longer recognizable as individual fibers. Some details of manufacturing and bonding method are presented in Fig. 1. Morphologically, the TSPCU leaflets consisting of nanofiber nonwovens showed a smooth surface and slightly sticked together. The fiber diameters of the nanofibrous leaflets were 870 ± 140 nm of valve A14, 720 ± 140 nm for valve B12, and 770 ± 110 nm for valve B13. The leaflet thicknesses were 100 ± 15 µm for valve A14, 90 ± 10 µm for valve B12, and 146 ± 15 µm for valve B13.

### Performance in static operation

**Fig. 2:**
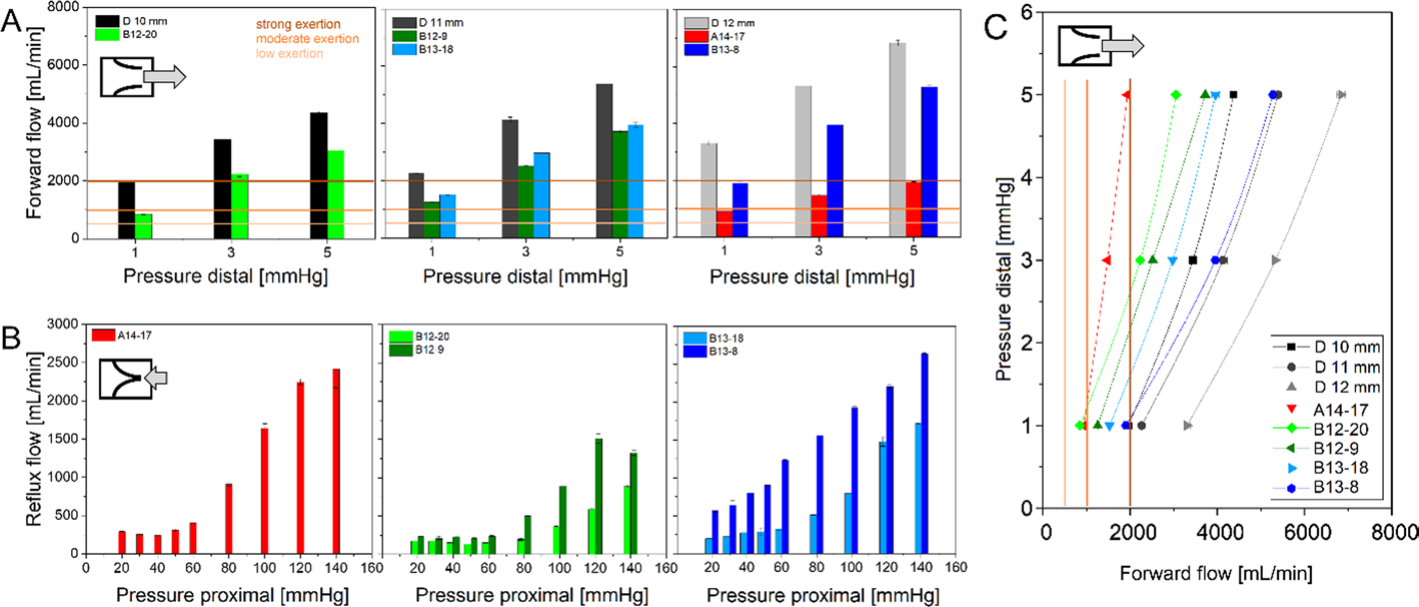
Static test results*.* **A** Open valves (under distal pressure) with inner attachment diameter of D 10 mm (left), D 11 mm (mid), and D 12 mm (right), black/gray color: attachments without valve, red: valve version A14, green: version B12, blue: version B13, **B** closed valves (under proximal pressure), version A14 (left, red), version B12 (mid, green), version B13 (right, blue), **C** comparison of pressure-flow characteristics (under distal pressure) of valves and attachments, *n* = 3 measurements each. **A**, **C** Indicator lines for low (light orange), moderate (orange) and strong exertion (dark orange)

The valve performance was tested for both directions, open (forward flow) and closed valves (reflux). Water pressure equivalent to 1, 3, and 5 mmHg for forward flow, as well as 20 up to 140 mmHg for reflux was achieved by adjusting water levels of distinct height (see Fig. 6). Maximum possible flow values were measured using attachments without valves. Valve A14–17 allowed about 1/3 of these maximum flow to pass through, with valves of version B approximately 2/3 of maximal flow values were observed, see Fig. 2A, C. The results of reflux are displayed in Fig. 2B.

Valve A14–17 revealed a reflux less than 310 mL/min for the physiological pressure range of 30–50 mmHg. Considerably increased reflux was observed at a pressure of more than 80 mmHg with a maximum of 2420 mL/min at 140 mmHg. After the experiment, the valve was found to be deformed and tilted, see Figure S16. The valves of version B12–9 and B12–20 revealed a reflux of less than 225 mL/min and 180 mL/min for the physiological pressure range, respectively. Considerably increased reflux was observed at a pressure of more than 80 mmHg (B12–9) and 100 mmHg (B12–20). A maximum reflux of 1510 mL/min (B12–9) and 890 mL/min (B12–20) resulted. The valve B13 revealed a reflux flow less than 905 mL/min (B13–8) and 290 mL/min (B13–18) for the physiological pressure range, respectively. Reflux was considerably increased at a pressure of more than 80 mmHg (B13–18). In contrast, for B13–8 a pressure proportional increase of reflux was observed with a maximum of 2635 mL/min.

### Performance in pulsatile operation

**Fig. 3:**
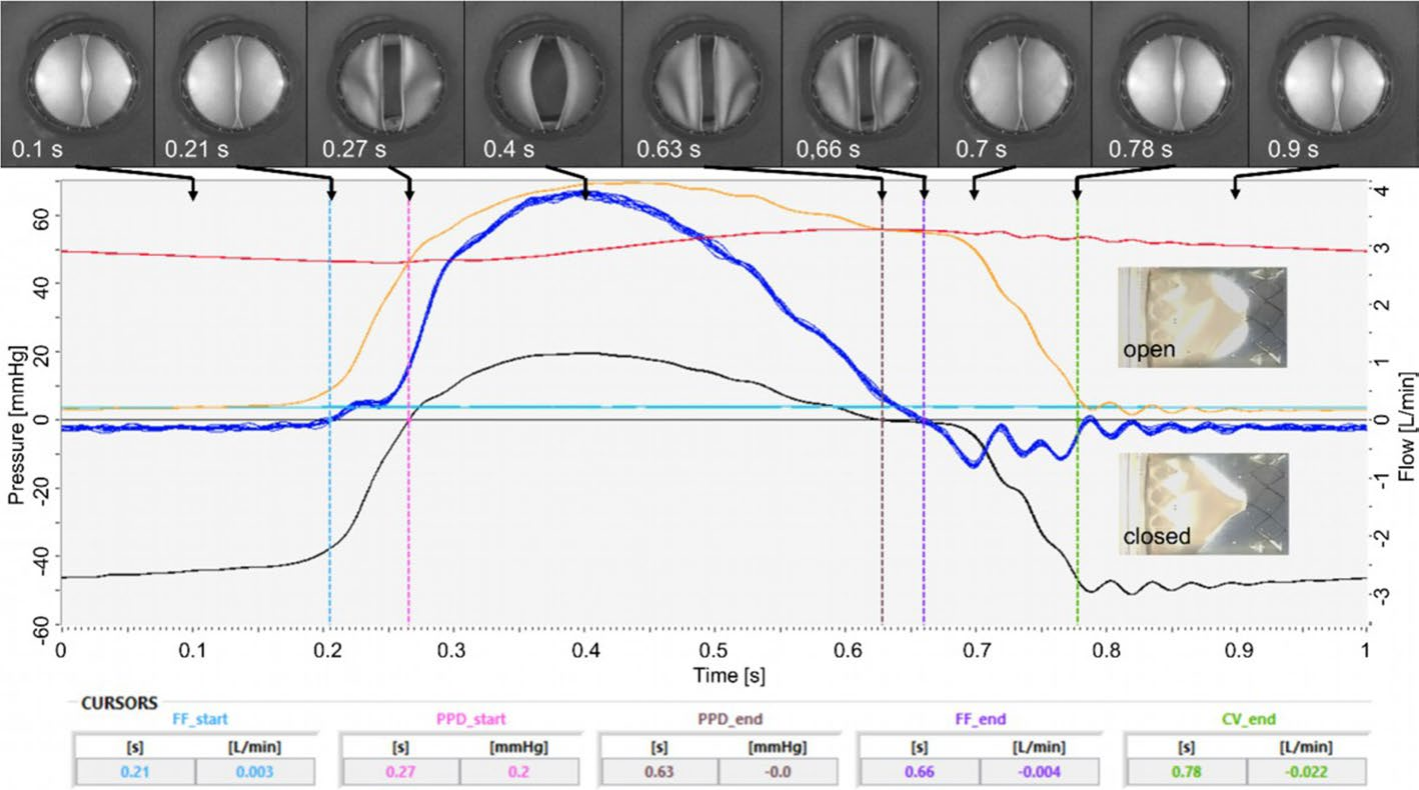
Pulsatile experiment, valve version A14–17, 60 bpm, 1 L/min, 50 mmHg. Curves (*n* = 10): red—pressure proximal to the valve, yellow—pressure distal to the valve, black—difference pressure, dark blue—flow, light blue—pre-pressure at water reservoir, cursors: FF—forward flow, PPD—positive pressure difference, CV—closing volume, Images: from proximal inline camera at points in time as specified, and photographs from outside of open and closed state

To illustrate the valve performance at a representative working condition, a typical operating pressure of 50 mmHg imitating moderate physical exertion is compared here for both valve versions. The complete data series mimicking different exertions are provided in supporting information 4. Experimental results of pulse duplicator testing of valve A14–17 at 60 bpm revealed valve opening at 0.21 s, forward flow until 0.66 s, maximal flow at 0.4 s of about 3.5 L/min, and valve closing at 0.7 s with some post-pulse oscillation, see Fig. 3. A total reflux of 1.75 mL per cycle was measured. The calculated effective orifice area (EOA) was 0.27 cm^2^, i.e. 24% of attachment cross-sectional area.

**Fig. 4:**
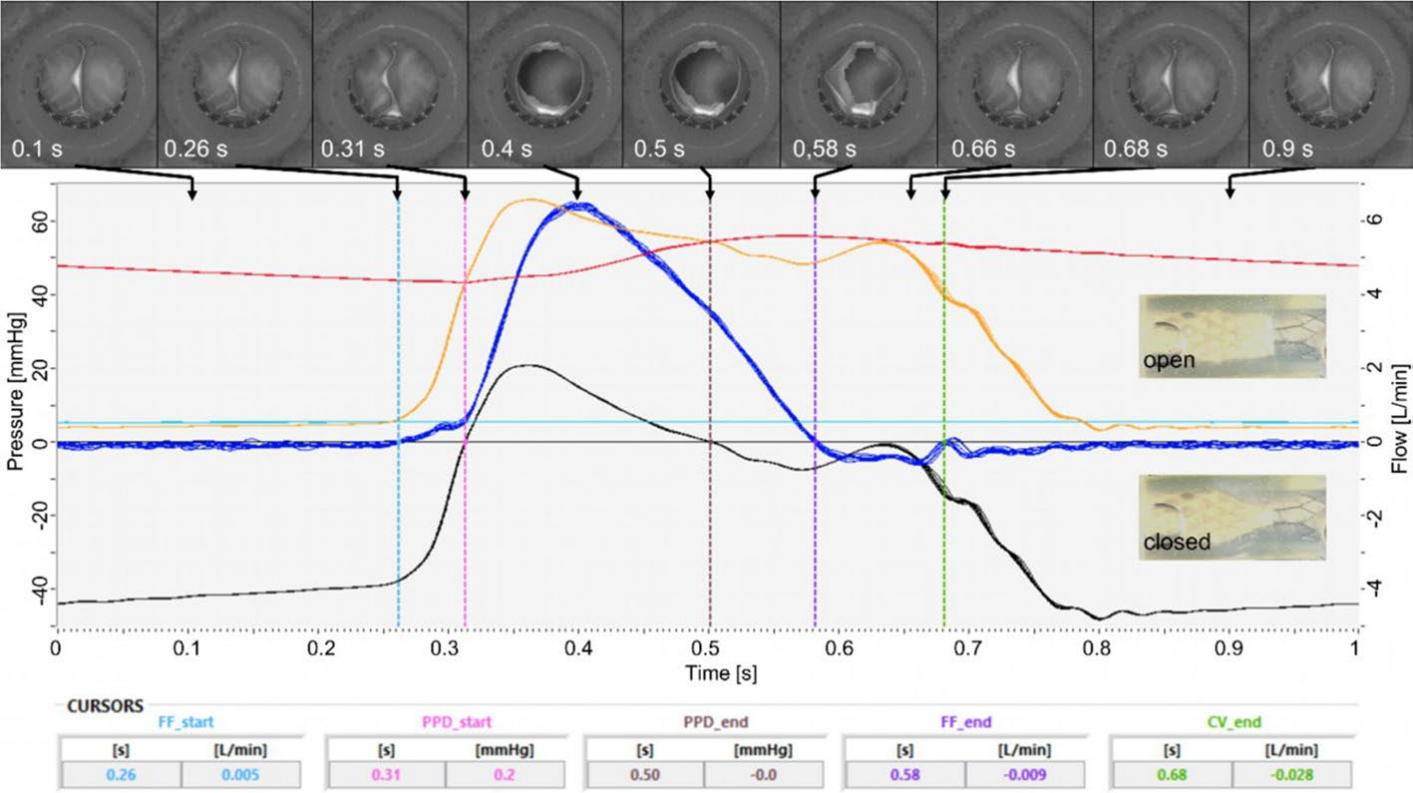
Pulsatile experiment, valve version B12–9, 60 bpm, 1 L/min, 50 mmHg. Curves (*n* = 10): red—pressure proximal to the valve, yellow—pressure distal to the valve, black—difference pressure, dark blue—flow, light blue—pre-pressure at water reservoir, cursors: FF—forward flow, PPD—positive pressure difference, CV—closing volume. Images: from proximal inline camera at points in time as specified, and photographs from outside of open and closed state

Pulsatile testing of valve B12–9 at 60 bpm revealed valve opening at 0.31 s, forward flow until 0.58 s, maximal flow at 0.4 s of almost 6.5 L/min, and valve closing at 0.66 s, see Fig. 4. A total reflux of 1.68 mL per cycle was measured. The calculated EOA was 0.49 cm^2^, i.e. 51% of attachment cross-sectional area.

**Fig. 5:**
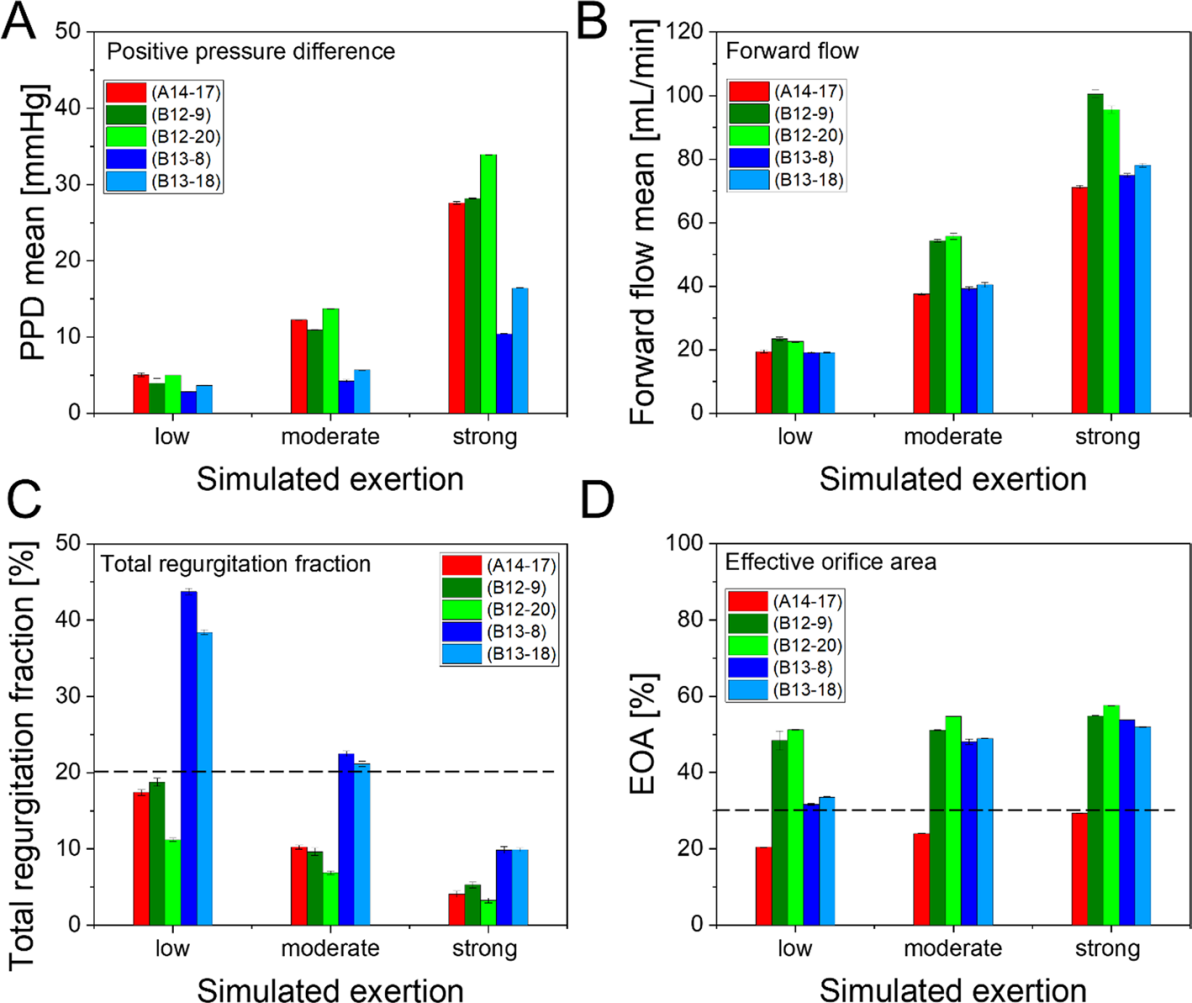
Representative pulsatile performance data at operating pressure of 50 mmHg. Simulated exertion as indicated: low (0.5 L/min at 40 bpm), moderate (1 L/min at 60 bpm), strong (2 L/min at 80 bpm), tests: *n* = 10, each. **A** positive pressure difference (PPD), **B** forward flow, **C** normalized total regurgitation fraction, dashed reference line at 20% indicating acceptable reflux [56], **D**: effective orifice area (EOA) normalized to circular cross section, dashed reference line at 30% as recommended minimum value [13]

Characteristic flow parameters and three simulated physiological conditions at operating pressure of 50 mmHg are summarized in Fig. 5. Similar PPD values were consistently observed for versions A14 and B12 across all simulated exertions. The leaflets opened and closed simultaneously on both sides evenly and symmetrically for both versions (see supporting information 5, especially Figure S17 and Figure S18). In contrast, version B13 required approximately half pressure at simulated moderate and strong exertion. The mean forward flow of version B12 was noticeable higher than versions A14 and B13, which revealed similar values. The total regurgitation fraction decreased with increased simulated exertion, for all tested valves. A significant higher regurgitation fraction was observed for valve B13. Version A revealed an increasing EOA of about 20–30% of circular cross section with rising simulated exertion. The EOA of version B12 also slightly increased with twice as large values (compared to version A14) of 50% to almost 60%. In difference, the EOA of version B13 increased from 30% to about 50% with rising simulated exertion.

## Discussion

### Physiological load of standing upright is simulated by static testing

Assuming that the EOA at static flow conditions is comparable to pulsatile operation, the difference in forward flow ratios of empty and valve loaded attachments, between the valve versions can be explained. For proximal pressure up to 60 mmHg all valves (except B13–8) revealed nearly constant reflux, over 80 mmHg the reflux considerably increased with pressure, which indicates functionality with some safety reserve. The proportionally to pressure increasing reflux of version B13–8 can be seen as hint for insufficient oversizing and leakage, despite proper valve function in pulsatile operation. Consequently, 8% oversizing revealed as insufficient sealing in this experimental setup. Hence, version B revealed functional with safety reserves in the physiological pressure range and beyond, if sufficiently oversized. A larger stent diameter allows slightly increased flow at a given vessel diameter (versions B), but the EOA has a significantly greater influence on the flow (version A vs. B).

Version A revealed limited performance due to significant higher flow resistance as a fluid dynamic deficit, which may be a potential risk factor for thrombosis. However, the static condition is more theoretical than practical, muscle activity is always necessary for upright standing. Small amounts of blood of different volumes are continuously pushed towards the heart by the muscle pump, which in reality means semi-pulsatile valve operation.

### Physiological load of walking is simulated by pulsatile testing

All valves are functional in acute testing conditions showing an almost constant operating behavior over the tested pressure and flow ranges, see Figures S1–S15, there is no concerning drifting out of line. The mean PPD of version B13 at simulated moderate and strong exertion was almost half the values of versions A14 and B12 (with similar forward volume per cycle), this indicates significantly less flow resistance or longer time duration. For versions A14 and B13 similar mean forward flow was observed, but with half pressure for B13, i.e. version B13 is of less flow resistance. Due to similar PPD values (A14 and B12) the higher forward flow indicated less flow resistance of version B12. The flow resistance of A14–17 is more than double the value of B12–9, which can be explained by different EOA. If the standard for heart valves is considered as a guideline, reflux in the range of 10–20% are described as acceptable (Table S2) [56]. The tested constellations except B13–8 fulfil this requirement. For natural saphenous veins a closing pressure of 2–5 mmHg at flow of 12–32 mL/min is reported [57]. Both versions appear applicable under physiological conditions. However, without any flow or pressure, the valves of both versions are slightly open and allow slow blood flow without considerable resistance, e.g. horizontally lying person.

### Risk of valve displacement

Valve migration was not observed in the experiments presented here. As the inner surface and the compliance of a real vein differ from 3D printed material, no valid statement can be drawn. Due to pressure difference within the valve, a force is generated in the axial direction, which the implant has to resist to avoid displacement. Nevertheless, rough estimation of axial force can be calculated with circular cross section area and proximal pressure for the closed valve. In forward direction, with increasing valve opening the axial forward force decreases, a suction effect due to the wake turbulence may also pull on the valve. Additionally, due to irregular leg movements, mass inertia of blood, and hydrodynamic conditions maximal axial short-term forces of about some Newton are realistic. Radial force distribution of oversized stents is not uniform due to anatomical environment or possibly uneven calcification [58]. However, recommendations for avoiding valve displacement require complex considerations including axial force, radial force, surface properties, and the anatomical–biological environment.

In contrast to the arterial system, vein diameter is not constant in different load situations and due to postural changes of the body, causing permanent changes in hydrostatic pressure and systemic conditions. Therefore, comparably high oversizing and a high compliance is essential for venous stent-based implants, to accommodate to the vessel diameter changes and achieve secure fixation. To avoid migration of the stent in the vein, oversizing of 1–4 mm (10–20%) is recommended [19, 59]. In general, veins show lower maximum stresses and Young’s moduli, but higher maximum strains compared to arteries, indicating that the thin venous wall can tolerate more extensive dilatation without rupture [60–62]. Nevertheless, permanent high overexpansion of the venous wall should be prevented, as it could affect neointimal growth [63]. High radial force can cause inflammatory response and fibrosis [59].

Implant migration or tilting can also be prevented by surgical suture or additional elastic stent elements. One further option is the utilization of barbs, which are for example used for inferior vena cava (IVC) filters [64], these are not further discussed here.

### Compliance with general recommendations

Considering the general recommendations derived from literature, following respects can be stated. Both versions are designed to being crimped for minimal invasive implantation. Secure fixation should be ensured, but permanent overexpansion of the vein wall should be prevented. The bicuspid design mimics blood flow similar to natural valves, version A causes higher flow velocity and thus washout of region behind leaflets, version B allows a high orifice area with less dead space. The orifice of an open venous valve is always elliptical, never circular [57], hence, natural flow conditions are achieved when the valve is not fully open. These are theoretical considerations, monocuspid valves are also under development, which imitate natural blood flow less conforming. Tilting and deformation occurred with the valve of version A below 80 mmHg in both scenarios (static and pulsatile), which could be prevented by the double strut of version B, no displacement was observed here. Manufacturing leaflets from material of the group of polycarbonate urethane is promising to us in regard of biocompatibility and thrombosis triggering, but in vivo tests were not performed in this study. Reflux was observed in the range of 1–4 mL/min in physiological pressure range at pulsatile testing, which is less than 10% of forward flow in most cases and fits mainly the recommended range. Valves of both versions open at a pressure gradient below 5 mmHg relating to natural conditions. The ratio of implant length to diameter is controversial, a short implant is less burden for the vein wall and easier to handle, but is of increased tendency for tilting. A long implant shows less tendency for migration, but has to withstand bending [60]. That ratio is for version A 1.6, and for version B 4.6. Version can be designed differently by adapting the number of cell (rhomboid) rings and changing the length of double struts (keeping the mid part) without impairing its performance. The EOA of version A14–17 revealed less than the recommended area of 30% of vessel cross-sectional area [50], valves of version B fulfil this requirement. The long-term performance of the valves was not yet investigated, the focus was on functional testing prior enduring studies. The long-term requirements are estimated as followed: when a hypothetic patient slowly walks 1 h a day with a cadence of 40 steps per minute, the valve performs 2400 cycles per day or 0.9 million steps per year, Sathe et al. recommend durability for at least 500.000 cycles [35].

Depending on the stage of CVI disease, the vascular situation can be diverse in terms of venous dilation and deformation. An advanced therapy concept may comprise several implantation sites for valve prostheses. A possible therapy approach that takes into account superficial and deep implantation sites, in consideration of the hemodynamic paradox [7] as an example, could work with differently designed valves that are tailored to the respective conditions. For this advanced therapy, valve prosthesis of version A (shorter, more flexible) seem to be more suitable for peripheral system, and valves of version B (longer, more sturdy) are well suited for central veins. Summing up, the intended positive effects of version B have been confirmed in the experimental findings.

### Further aspects for the application of venous valve prostheses

The utilization of non-magnetic or non-metallic material is advantageous in order not to limit the spectrum of available diagnostic examinations or therapy options for a patient, e.g. MRI ("MR-safe" or "MR-conditional"), and radiothermy. Further, a concept for diagnosing the current state of the implant itself is also recommended, regarding to wear, aging, structural failure. This can be achieved e.g. through accessibility for ultrasound, radiopaque elements, sensing wires, or microelectronic components. Sonography is limited in metallic stents, but in the here presented design the leaflets are accessible. A prospective special biologized valve that prevents both neointima and intimal hyperplasia and improves the functionality of the valves could be achieved with tissue engineering, e.g. seeding with endothelial cells [18].

In addition, durability and crimpability for minimal invasive implantation of the whole implant will be investigated in forthcoming work. Further, studies on different stent materials (stainless steel, cobalt chrome alloys, or nonmetallic or polymeric material) are intended.

### Venous valve design recommendations

Considering the testing results, also concerning to generally accepted commendations of other authors, we derive design recommendations for prosthetic venous valves as listed below, see Table 1.

**Table 1:** Recommendations for new version of bicuspid venous valve design and testing conditions

	Parameter/aspect	Recommendation	Explanation based on our test results
Advances in similar valve designs	Stent oversizing	10–20% of vessel diameter	Vessel dilation should be considered 8% revealed as too small, 9% is the limit in tests without vessel deformation More than 20% may injure the vein wall [19, 59]
	Stent fixation	Additional elastic stent retaining structures	Prevention of dislocation E.g. hooked barbs may penetrate venous wall
	Stent structure	2–3 cell rings (e.g. rhomboid) at the top and bottom	Acceptable implant length Sufficient stability Single rings showed tendency to rocking motion
	Cell ring connector	Double struts between leaflet and upper cell ring	Increase stability Prevention of deformation and tilting
	Stent length/diameter	Ratio (L/D) more than 2	Prevention of tilting Shorter ring connection reduces implant length
	Stent sidewall	Filled with cells (e.g. rhomboids)	Prevention of inward bulging of wall material Version A vs. version B, see also Fig. 1
	Effective orifice area (EOA)	Large (maximum)	Less flow resistance Larger than 30% of the vessel cross-sectional area [13, 50]
	Leaflet fixation	Fixation of leaflet in cells (rhomboids) of sidewall	Prevention of leaflet sliding down Improved sealing to vessel wall
	Leaflet material	Flexible, biocompatible, antithrombotic, long-term stable, non-biological origin	Artificial (synthetic) material has no variations in quality, texture, durability, etc. compared to natural material Less complexity in testing (in vitro, in vivo)
Specific test parameters	Pressure range	Runing up to at least twice the physiologically expected pressure values	Discovering hidden weaknesses Safety reserve
	Opening/closing pressure	Opening and closing at low pressure difference (below 5 mmHg)	Performance close to natural valves
	Flow range for vessels (Ø > 10 mm)	Capability to operate at flow of 2 L/min	No failure under high operational load
	Frequency	60 bpm (minimum)	Simulation of realistic exertion scenario
	Reflux	< 20% of forward flow (pulsatile operation)	Sufficient functionality

### Limitations

From valve version A only one size was available, due to the aforementioned deficiencies and the consequently design of version B, no further sizes of version A were manufactured. The influence of leaflet thickness cannot be clearly distinguished from other design effects, in consideration the other findings a subordinate role is presumable. In static and pulsatile conditions, the whole flow was measured, discriminating perivalvular leak fraction from the flow fraction through the valves was not possible. Based on the inline camera video recordings at high pressure levels, no gaps or incomplete radial stent apposition were apparent, so that only minor influence of perivalvular leakage was assumed. The long-term performance of the valves has yet to be investigated, as the focus of the present study was on functional testing prior to enduring studies. The crimpability of version B has not yet been investigated to determine whether the leaflet structure has been damaged, but this will be done in future work. The inner surface of a natural vein differs from attachments made of 3D-printed material, and the compliance of the vein wall depends on the tension of smooth muscles. Therefore, statements about oversizing are of limited validity.

## Conclusion

In this study, a modified venous valve concept was comparatively compared to the original design by performance testing under static and pulsatile flow conditions. The valve leaflets were fabricated of electrospun TSPCU nanofiber nonwoven, due to favorable biocompatibility and simple processability. It could be shown that the ES parameters can be adapted to both setups of versions A and B, without any particular advantage of one concept over the other. However, version B is far easier to produce, eliminating the elaborate process step of nonwoven transfer from an ES mold, while also improving leaflet thickness homogeneity and maximizing EOA. Regarding stent design, version B improved stent anchoring and leaflet fixation. In terms of material selection, the approach of using low Shore hardness TSPCU material could be substantiated.

Both valve versions performed under different simulated physiological conditions, whereas version A revealed a tendency to reach its limits in the situation of higher operational loads earlier. It can be summarized that the design modifications in version B demonstrated the intended positive effects on valve performance, but the modified stent design is initially a test platform rather than a final implant, it points the way to more suitable venous valve implants. The evaluation demonstrate the impact of design changes identifying key elements for future implant developments. Specific design and testing recommendations were drawn.

Future work will focus on long-term functionality, which will be investigated by endurance testing and comprehensive biocompatibility testing of various leaflet materials in vitro and in vivo is intended.

## Materials and methods

### Bicuspid prosthetic venous valves designs

The initially developed design is in the following named “version A” [55], the modified design is named “version B”. In light of the aforementioned requirements, two bicuspid valve concepts with valve leaflets made of thermoplastic silicone polycarbonate urethane (TSPCU) nanofiber nonwoven material were investigated. The valve leaflets are attached to nitinol stents consisting of two cell rings (rhomboid) and a leaflet support in between the cell rings, see Fig. 1. Version A consists of two cell rings with ten rhomboid-shaped elements, which are connected by two arched struts in between. The leaflet structure is attached to the lower ring, and the commissure of the leaflet matches the support structure, so the valve remains in its shape. The results of preparatory experimental studies on version A have encouraged the development of version B, which modifications are following:

- The rhomboid rings consist of three rows each instead of one, to avoid rocking motion.
- The side walls are filled with rhomboids for three reasons: (1) fixing the leaflets to prevent migration from stent attachments, (2) prevent inward bulging, (3) improved sealing to the vessel wall.
- Double struts were added between leaflet and upper rhomboid ring to improve stability and prevent tilting. In neutral position, the valve leaflets are to be slightly open.
- Application of leaflets with a cylindrical shape and a diameter according to the stent diameter for three reasons: (1) fully open cross-sectional area with lowest possible flow resistance, (2) no mold for electrospinning is needed, (3) possibility of using simple tube-shaped wrought material valves, which are not directly electrospun on the stent.

### Fabrication of testing specimens

#### Stent samples

Stent A was designed using the CAD software Creo Parametric 6.0.3.0 (Parametric Technology Corp., Needham, MA, USA). Stent B was designed in Fusion 360 2.0.17721 (Autodesk GmbH, München, Germany). The stents were manufactured from a nitinol tube with a diameter of 5 mm and a wall thickness of 0.24 mm (Euroflex GmbH, Pforzheim, Germany). The stents were laser cut using a StarCut Tube system (Coherent Corp., Saxonburg, PA, USA) with a pulsed laser source Monaco 1035-80-60, frequency 500 kHz, pulse duration 300 fs, pulse energy 10 µJ, argon atmosphere with supply pressure 15 bar, and cutting velocity of 1.5 mm/s. Stent A was expanded in two steps with increasing diameter to 14 mm in a convection oven KLS 05/13 (Thermconcept GmbH, Bremen, Germany) with an annealing temperature of 512 °C for 15 min at each step. The stents of version B were expanded in three steps to final diameters of 12 and 13 mm, respectively, with 8 min annealing time per step. A polishing step followed afterwards.

#### Valve leaflet samples

A homogenous polymer solution of 7.5wt% TSPCU (Shore 80A) was obtained by dissolving in chloroform (CHCl_3_), *N,N*-dimethylformamide (DMF) and 2,2,2-trifluoroethanol (TFE), (ratio of 3/1/1). All polymers, chemicals and solvents were used as received. In brief, electrospinning was performed using an experimental spinning setup equipped with a single jet capillary emitter (G21 cannula) and a rotating mold or cylinder, see Fig. 1. Following parameters were set: working distance of 160 mm, rotation of 4000 rpm, flow of 1 mL/min, linear traversing motion, room temperature. A high voltage of 6 kV (collector) and 22 kV (emitter) was applied and processing time was fixed at 7200 s. A layer thickness of 100 µm was targeted for both versions. For valve version A, CNC machined aluminum molds (Haas Automation, Inc–CNC Machine Tools, Oxnard, CA, USA) were coated with electrospun TSPCU nonwoven as described in [65]. The stents of version B were directly coated on the metal cylinder.

#### Assembly of valves

The electrospun nonwoven was removed from the mold and transferred to stent A, or removed from the cylinder with stent B. For fixing valves of both versions on the stents, the protruding part of the nonwoven was turned inwards and spot-welded in all rhomboids using an in-house developed device [66], see Fig. 1.

The nonwoven thickness (*n* = 10, each) was measured using a dial gauge Mitutoyo 543–394B with associated stand (Mitutoyo Corporation, Kawasaki, Japan).

The fiber morphology of leaflet and welded points were investigated using a scanning electron microscope (SEM) QUANTA FEG 250 device (FEI Company, Dreieich, Germany) operating in high vacuum and 10 kV voltage, using an Everhart–Thornley detector (ETD) in secondary electron mode. The nonwoven samples were fixed on aluminum carriers using conductive carbon pads. Gold sputter coating was achieved using an AGAR Sputtercoater (Agar Scientific Ltd, Stansted, Essex, UK). The fiber diameters were measured for quality control applying the GIFT-method [67, 68].

#### Testing fixtures

Attachments, which are simplified representations of vessels for experimental tests, were designed in three sizes with inner diameters (D10 mm, D11 mm, and D12 mm) using CAD software Fusion 360. Digital light processing (DLP) technology was used to create annular attachments of photopolymeric resin Ultracur3D FL 300 (BASF 3D Printing Solutions GmbH, Heidelberg, Germany) on an Asiga Pro 4K45 printer (Asiga, Alexandria, Australia), for details see supporting information 1.

### Experimental test setup for performance testing

For preconditioning and removing of potentially existing solvent residues the assembled valves were stored in ultrafiltrated water at room temperature for at least 2 days before testing. Under immersed conditions, little material shrinking was observed. Before testing, the valves were crimped by hand at 0 °C in ice water, then implanted in the attachments. The test configurations are introduced in Table 2.

**Table 2:** Test configurations, abbreviations for tested experimental arrangements specify the valve version, stent size, and the oversize in millimeters (O.S) resulting from the attachment, and oversizing percentage in brackets

Valve version	Stent diameter	Sample abbreviation in attachment (Ø_tube_) and oversizing (O.S)					
		Ø10 mm	O.S	Ø11 mm	O.S	Ø12 mm	O.S
A	Ø14 mm	X		X		**A14–17**	2 (17%)
B	Ø12 mm	**B12–20**	2 (20%)	**B12–9**	1 (9%)	X	
B	Ø13 mm	X		**B13–18**	2 (18%)	**B13–8**	1 (8%)

The valves were oversized by 1 or 2 mm in relation to the attachment diameter, as recommended in [19]. Regarding the experimental design, valve of version A (A14–17), available from previous work with a diameter of 14 mm has only been tested in a 12 mm attachment with 2 mm oversize. Preparatory studies revealed insufficient performance of valve A with 1 mm oversizing; hence, it was not pursued further for this work. In contrast, two version B stents (B12, B13) were tested in attachments with two different diameters each.

For performance testing of the different valves, static testing and pulsatile testing was done. An operating pressure of 50 mmHg was estimated for a valve in the thigh. In general, the tests were run up to more than double operating pressure (worst case) to be on the safe side and detect possible trends in performance.

#### Static testing

**Fig. 6:**
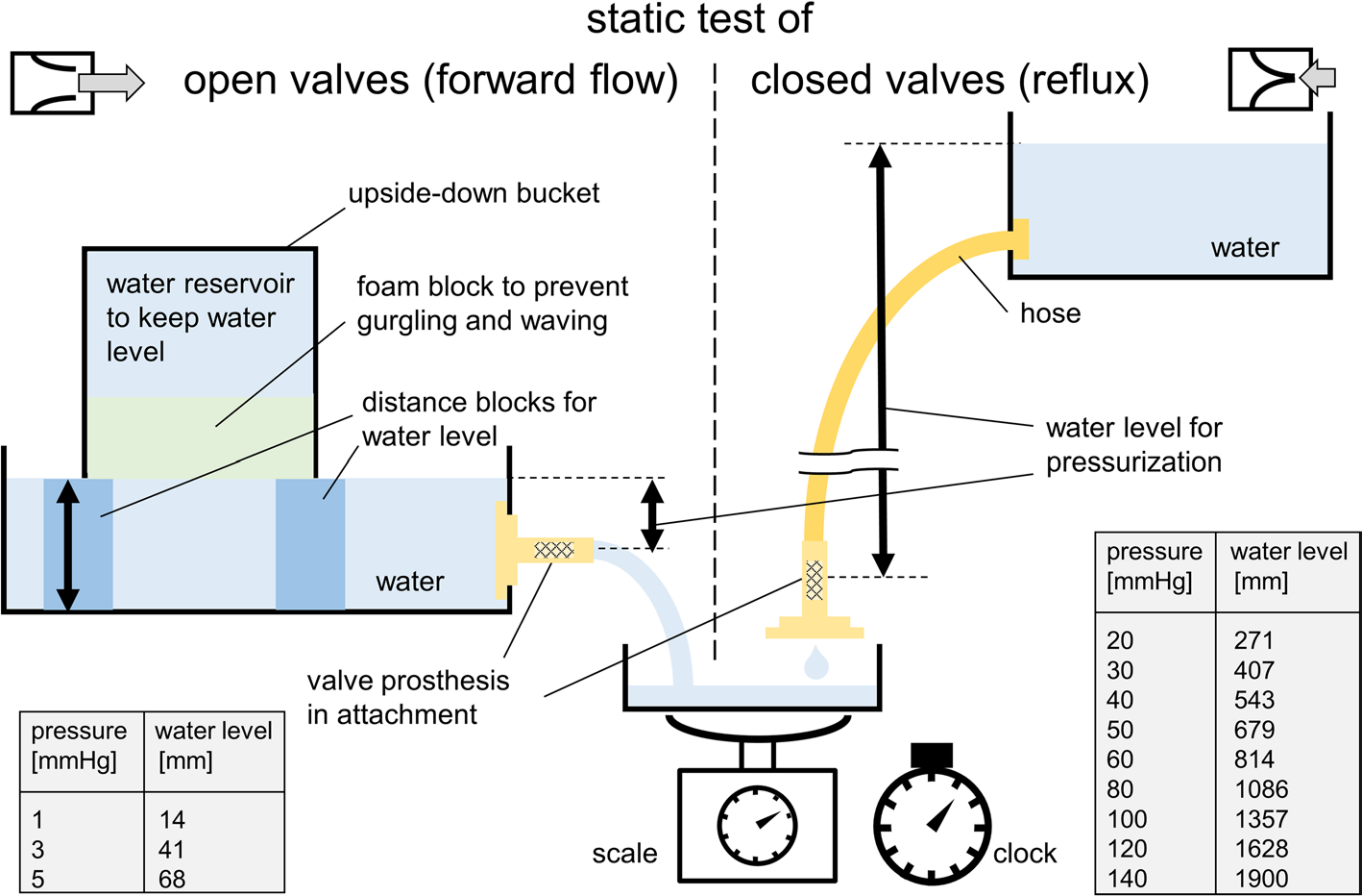
Static testing. Gravimetric measurement of water mass per time interval was performed for open valves (conditions: constant water pressure was generated by distance blocks under a reservoir) and closed valves (conditions: different height levels and a flexible hose between valve and water container were utilized)

The valves were implanted in different attachments, and testing was performed in forward and backward direction using water. The forward flow was measured at 1 mmHg, 3 mmHg, and 5 mmHg by gravimetric measurement of water mass, which flowed through the test specimens, at room temperature for a time interval of 30 s using a scale Sartorius MSE4202-000-D0 (Sartorius AG, Göttingen, Germany). Additionally the flow through empty attachments without valves was investigated (*n* = 3, each). The reflux was measured at 20–60 mmHg in 10 mmHg steps, and 80–140 mmHg in 20 mmHg steps by gravimetric measurement of water mass (*n* = 3, each). The values for pressure-equalized water column were calculated using a density value for water and mercury of 998.21 kg/m^3^ at 20 °C [69], and the density of mercury (13,545.89 kg/m^3^) [70] respectively, see Fig. 6 and Table S1.

#### Pulsatile testing

**Fig. 7:**
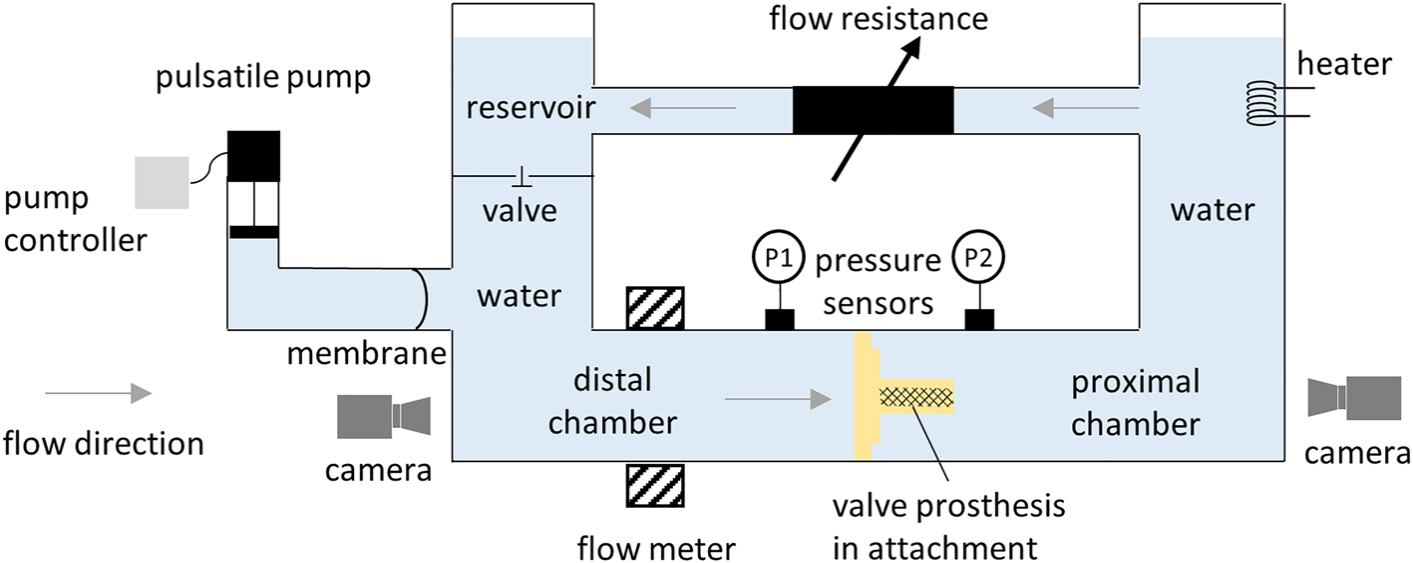
Pulse duplicator system (schematic drawing)

To investigate the fluid dynamics of venous valves under pulsatile, physiological, and pathological pressure and volume flow conditions, a fluid dynamic circulation model was used, shown in Fig. 7 and Figure S16, for details see supporting information 3. The test series were performed with flow rates of 0.5 L/min at 40 bpm to imitate slow walking or low exertion, 1 L/min at 60 bpm for moderate exertion, and 2 L/min at 80 bpm for strong exertion. The test setup, initially designed for testing aortic and mitral valves (HDTi-6000, Biomedical Device Consultants and Laboratories, Wheat Ridge, USA), consists of two fluid compartments coupled via a flexible membrane: the pump circuit and the test circuit.

Testing was performed using 0.9% saline at 37 °C. The valves were tested with ballasting pressure in ranges of 20–60 mmHg (A14) and 20–140 mmHg (B12, B13), increased in 10 mmHg steps. For simulation of different exertion conditions, frequencies and flow parameters of 40 bpm with 0.5 L/min each, 60 bpm with 1 L/min each, and 80 bpm with 2 L/min each were adjusted. The pressures distal and proximal to the valve were measured, as well as the flow through the valve loaded testing attachment. To achieve specific flow at definite proximal pressure values, the mutually affecting parameters of displacement volume of the pulsatile pump, as well as flow resistance were controlled.

## Supplementary Information

Supplementary Material 1

## Data Availability

No datasets were generated or analysed during the current study.

## Declarations

### Generative AI

Generative AI and AI-assisted technologies were only used in the writing process to improve the language and readability. Generative AI and AI-assisted tools were not used in data evaluation, images and artwork.
