# Supplementary material for: Transcatheter bicuspid venous valve prostheses: fluid mechanical performance testing of artificial nonwoven leaflets
Source: Biomed Eng Online. 2024 Nov 29;23:124. doi: 10.1186/s12938-024-01316-x (PMC11606079; doi:10.1186/s12938-024-01316-x)
Supplement: Supplementary file 1 — Supplementary Material 1 [file 12938_2024_1316_MOESM1_ESM.docx]

**Supporting Information**

**Transcatheter bicuspid venous valve prostheses: fluid mechanical performance testing of artificial nonwoven leaflets**

Andreas Götz* 1, Sabine Illner 1, Nicklas Fiedler 1, Julia Schubert 1, Jan Oldenburg 2, Heinz Müller 3, Wolfram Schmidt 1, Klaus-Peter Schmitz 2, Niels Grabow 1, and Kerstin Lebahn 1

1) Institute for Biomedical Engineering, Rostock University Medical Center, Friedrich-Barnewitz-Str. 4, 18119 Rostock, Germany

2) Institute for Implant Technology and Biomaterials e.V., Friedrich-Barnewitz-Str. 4, 18119 Rostock, Germany

3) CORTRONIK GmbH, Friedrich-Barnewitz-Str. 4a, 18119 Rostock, Germany

Corresponding author: Andreas Götz, andreas.goetz@uni-rostock.de

**Table of Contents**

1. **3D printing process**
2. **Pressure equivalent water levels**
3. **Pulse duplicator system**
4. **Results of pulsatile testing**
5. **Leaflet deformation**
6. **Standards**
7. **3D printing process**

All 3D printing processes were performed with an Asiga Pro 4K45 printer (Asiga, Alexandria, Australia) using digital light processing (DLP) technology. The LED projectors (UV-light, λ = 385 nm) xy-resolution in 4K-mode is 32 µm with a build platform of 122 x 68 mm² installed.

A base plate with a height of 0.400 mm was used as a supporting structure to ensure adequate built platform adhesion. The base plate was built using a layer resolution in z-direction (layer height) of 100 µm per layer, exposed for 30.210 s. Further layers of the parts were built using a layer height of 100 µm per layer, exposed for 8.934 s.

Attachments for experimental hydrodynamic testing were printed using flexible photopolymeric resin Ultracur3D FL 300 (BASF 3D Printing Solutions GmbH, Heidelberg, Germany). Burn-in layers and subsequent layers were sliced in 100 µm and exposed for 6.968 s and 1.675 s respectively.

Printed flexible attachments for hydrodynamic testing were washed twice for 5 min in an isopropanol (Sigma-Aldrich, Steinheim, Germany) filled ultrasonic bath and post-cured for 3000 flashes each side (6000 flashes in total) submerged in glycerol (Sigma-Aldrich, Taufkirchen, Germany). UV curing unit Otoflash G171 (NK Optik GmbH, Baierbrunn, Germany) was used for post-curing of all 3D-printed parts.

1. **Pressure equivalent water levels**

**Table S1: Pressure equivalent water levels**

| **Pressure [mmHg]** | 1 | 3 | 5 | 20 | 30 | 40 | 50 | 60 | 80 | 100 | 120 | 140 |
| --- | --- | --- | --- | --- | --- | --- | --- | --- | --- | --- | --- | --- |
| **Water level [mm]** | 14 | 41 | 68 | 271 | 407 | 543 | 679 | 814 | 1086 | 1357 | 1628 | 1900 |

1. **Pulse duplicator system**

Device: HDTi-6000 (Biomedical Device Consultants and Laboratories, Wheat Ridge, USA).

The pump circuit employs a piston pump (PD-1100, Biomedical Device Consultants and Laboratories, Wheat Ridge, USA), whose oscillating piston movements generate a pulsatile volume flow. The piston position is defined over time by a modified sinusoidal function, allowing the adjustment of the durations of systole and diastole as well as stroke volume and frequency. The system primarily consists of the pump chamber and three interconnected fluid chambers: the distal chamber, the proximal chamber, and the reservoir chamber, which hold 3 L, 7 L, and 11 L of test fluid, respectively. A valve between the reservoir and distal chambers prevent fluid backflow to the reservoir, thus enabling pressure increase in the distal chamber during piston advance. By adjusting the flow resistance and fill volume in the proximal and reservoir chambers, the mean system pressure and pressure amplitudes during pulsatile testing can be regulated. During piston advance, the test fluid flows through the venous valve model, with the volume flow measured immediately upstream of the model using a bidirectional in-line volume flow sensor (PXN25, Transonic Systems Inc., Ithaca, USA). The flow sensor was calibrated for 0.9% sodium chloride solution at a temperature of 37°C within the measurement range of −25 to 25 L/min. For these specifications, the absolute accuracy of the measurements is ± 4%. The kinematics of the venous valves were recorded using high-speed cameras (Basler acA1300-200um with ON Semiconductor PYTHON 1300 CMOS sensor, Basler AG, Germany). The high-speed cameras are equipped with fixed focal length lenses (50mm C Series Fixed Focal Length Lens f/2.0, Edmund Optics Inc., USA). This configuration produces images at a frame rate of 200 s^−1^ with a resolution of 1280 x 1024 pixels, thus allowing adequate characterization of valve kinematics. The cameras are synchronized with the piston pump. For pressure measurement up- and downstream of the venous valve, two identical relative pressure sensors (PendoTECH, Princeton, USA) are included. These differential pressure sensors have a maximum measurement deviation of ± 0.9 mmHg and a maximum relative deviation of 0.29% of the measured value within the range of 0 – 310 mmHg.

1. **Results of pulsatile testing**

The results of pulsatile test series are displayed in following bar charts, further explanations are included in each graphic.

Explanation of terms:

- Forward flow volume: forward flowed fluid volume, per cycle
- Closing volume: visualized in section 6 (Standards), per cycle
- Leakage volume: visualized in section 6 (Standards), per cycle
- Total regurgitation volume: sum from closing volume and leakage volume, per cycle


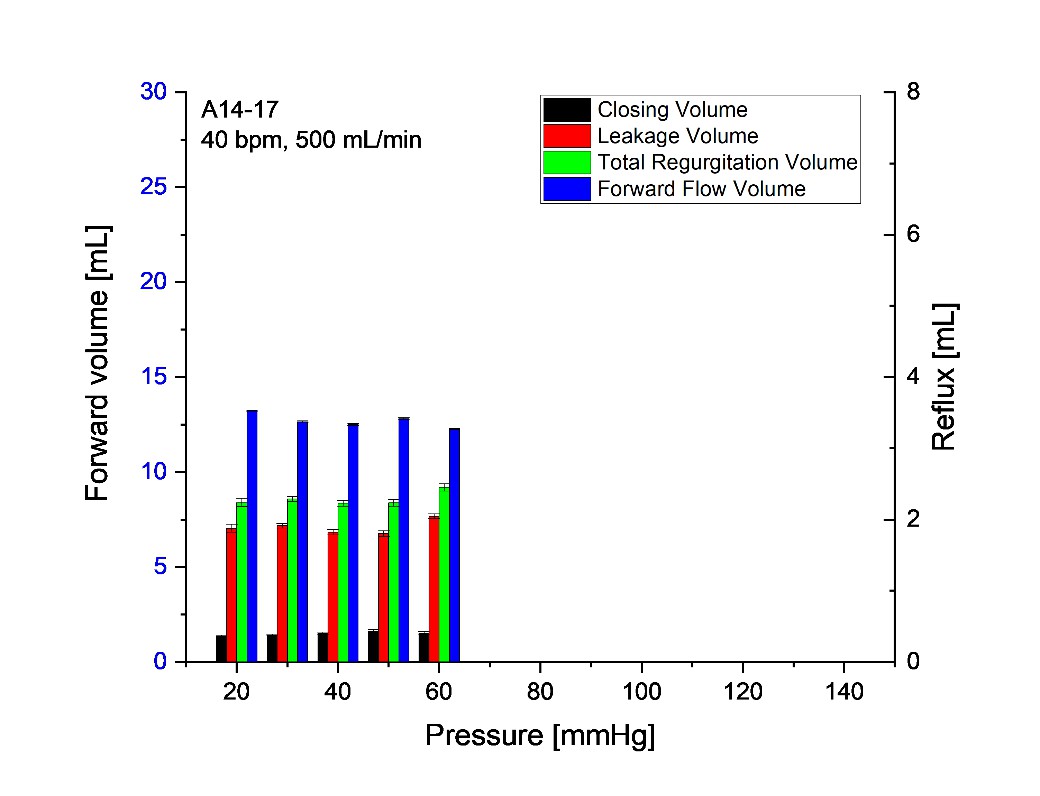


**Figure S1: valve A14-17, test series imitating low exertion**

**
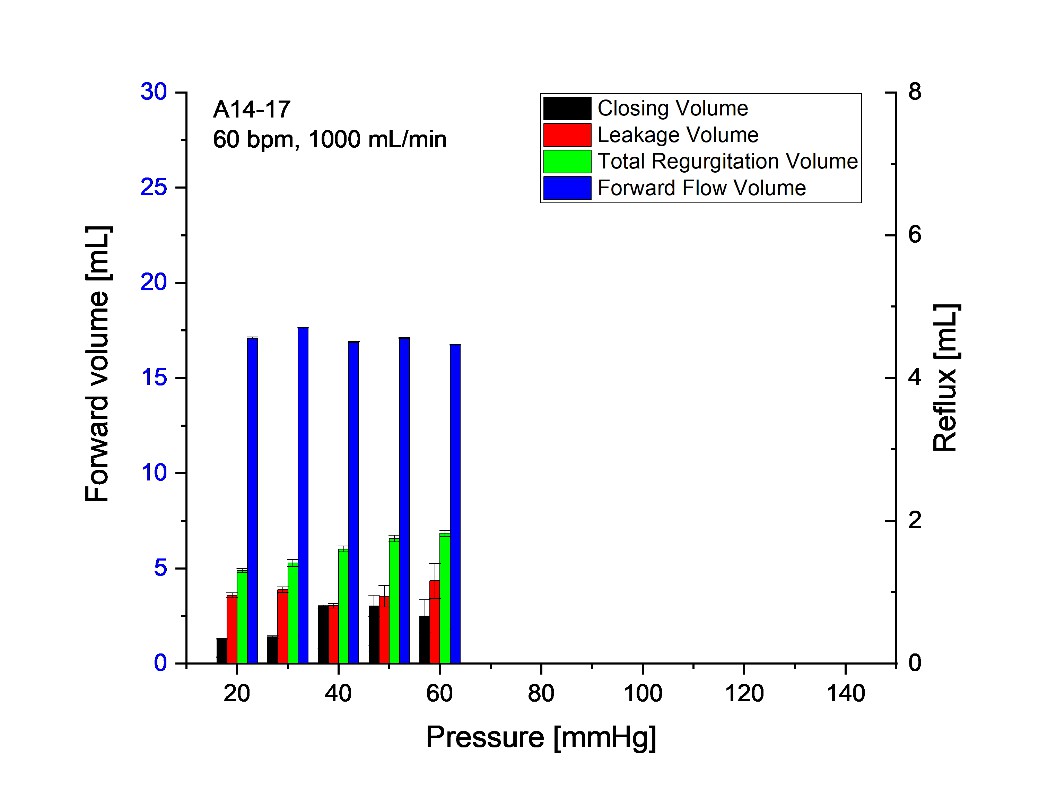
**

**Figure S2: valve A14-17, test series imitating moderate exertion**

**
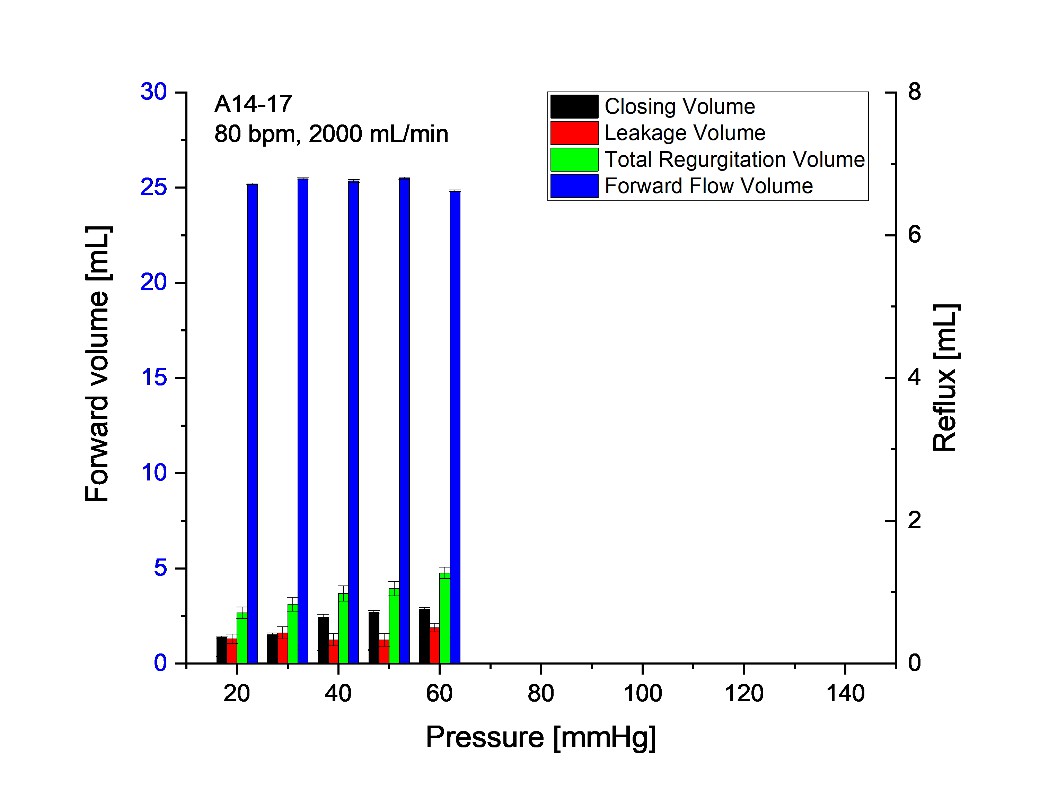
**

**Figure S3: valve A14-17, test series imitating strong exertion**

**
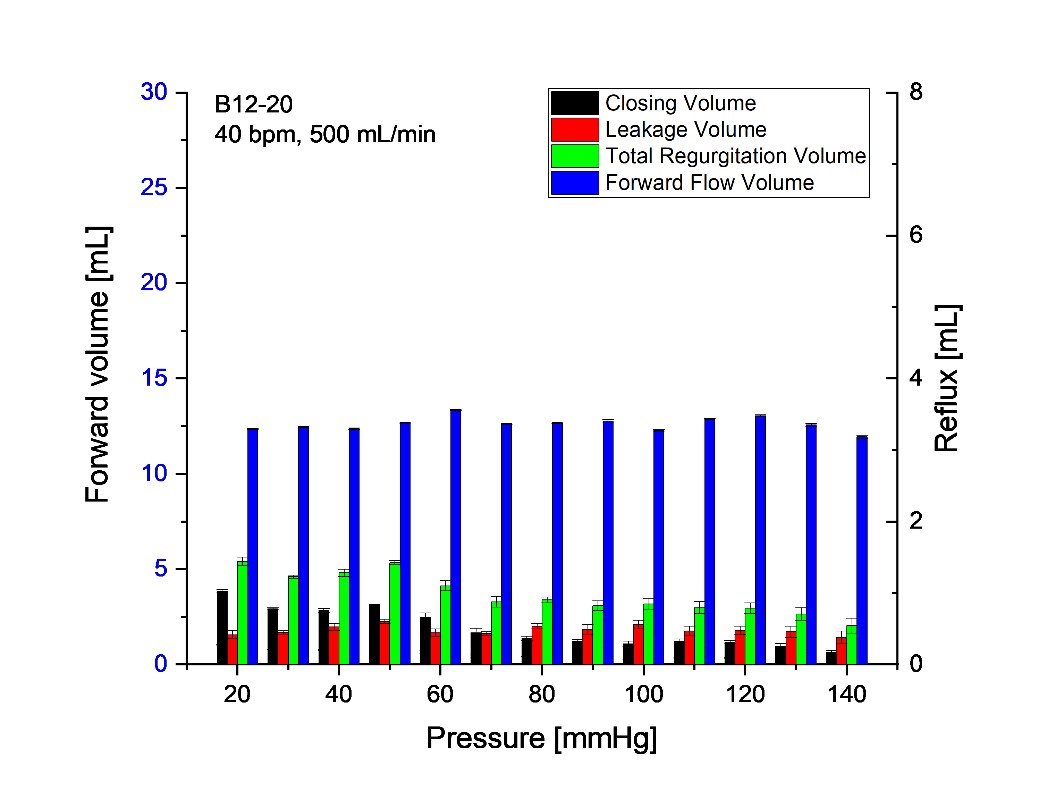
**

**Figure S4: valve B12-20, test series imitating low exertion**

**
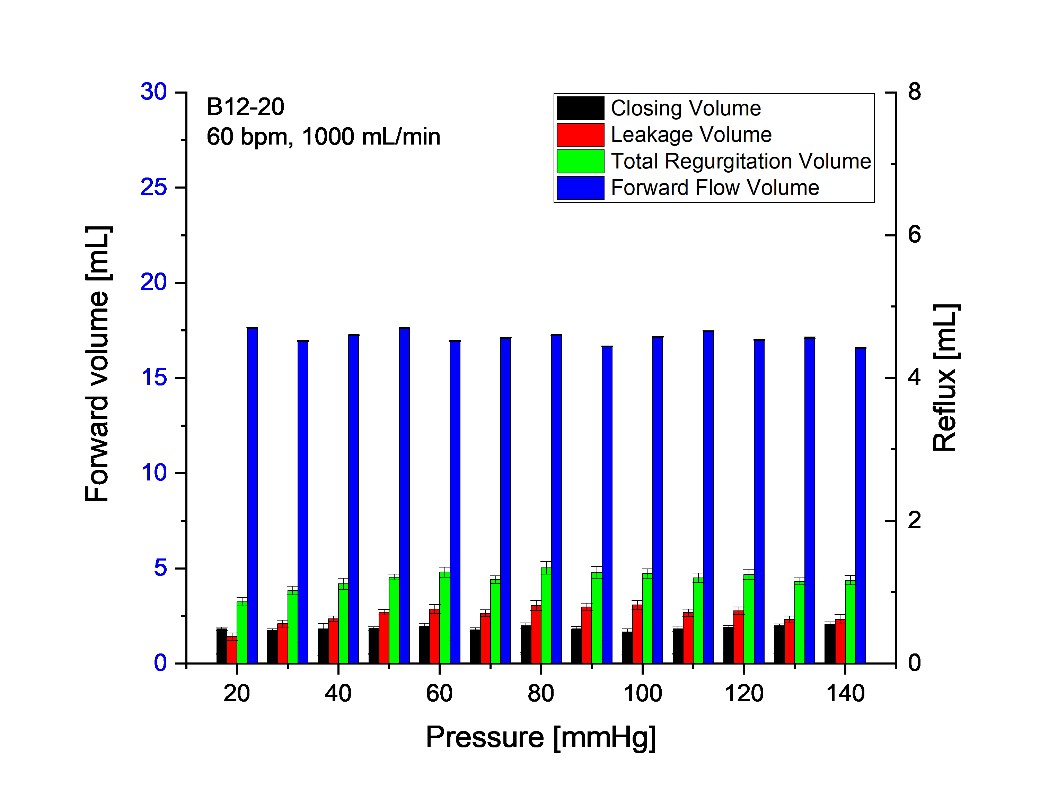
**

**Figure S5: valve B12-20, test series imitating moderate exertion**

**
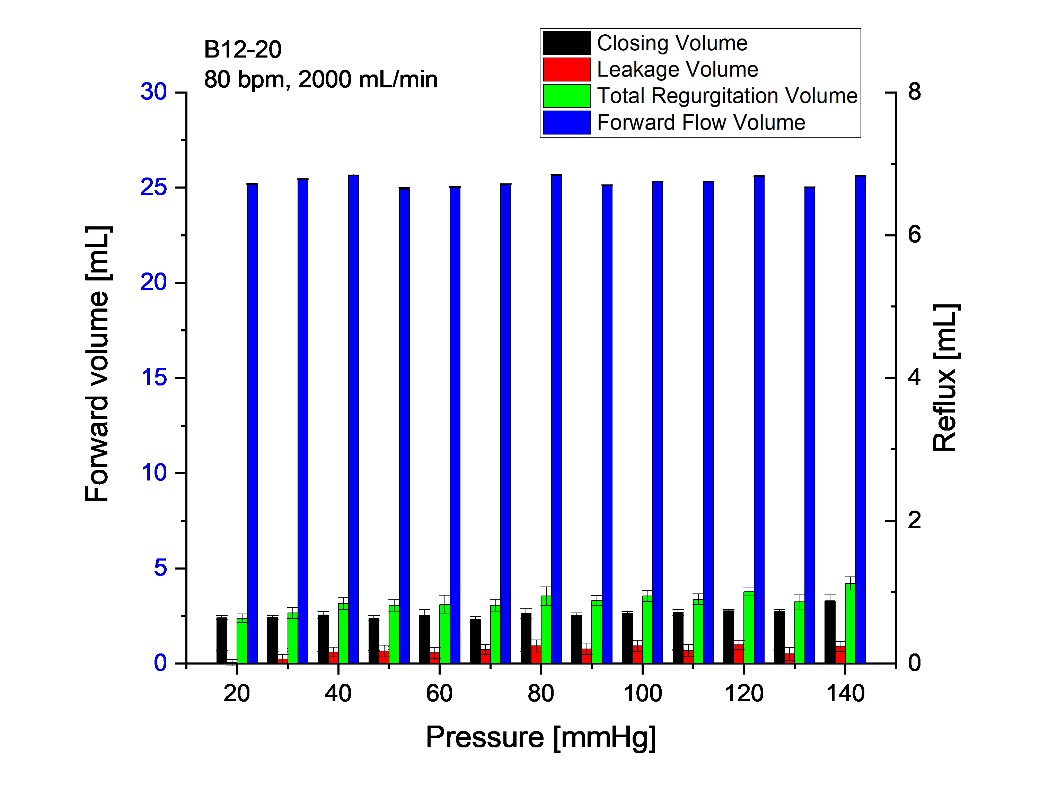
**

**Figure S6: valve B12-20, test series imitating strong exertion**

**
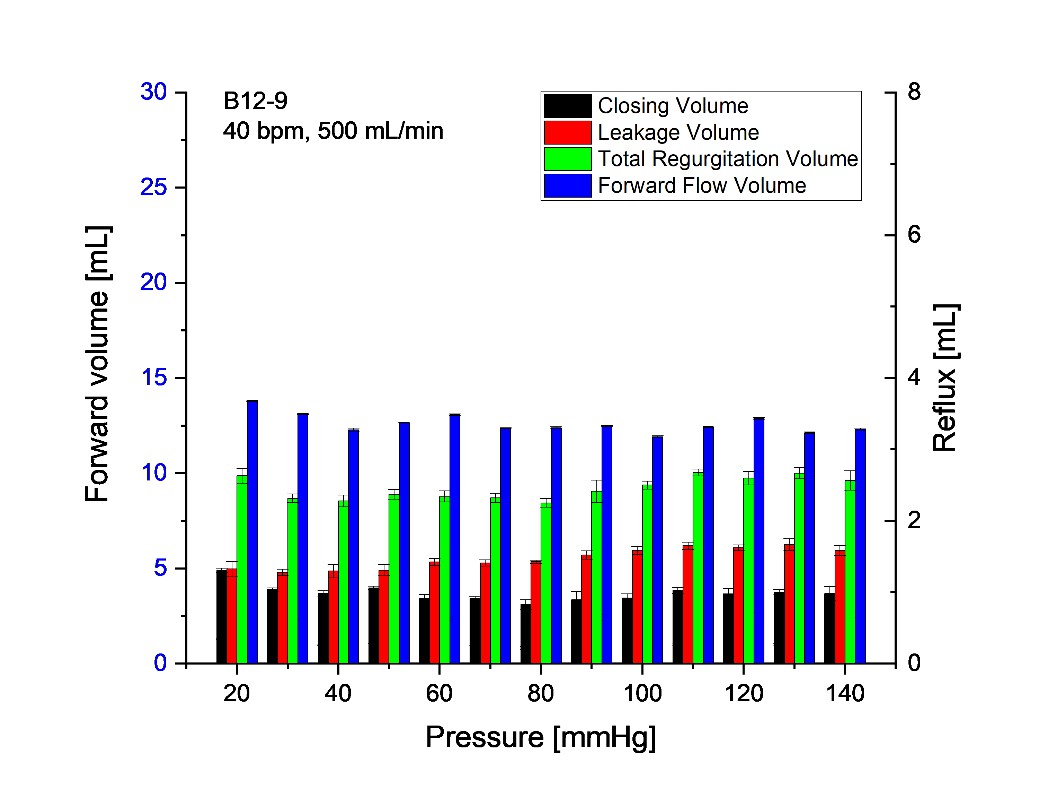
**

**Figure S7: valve B12-9, test series imitating low exertion**

**
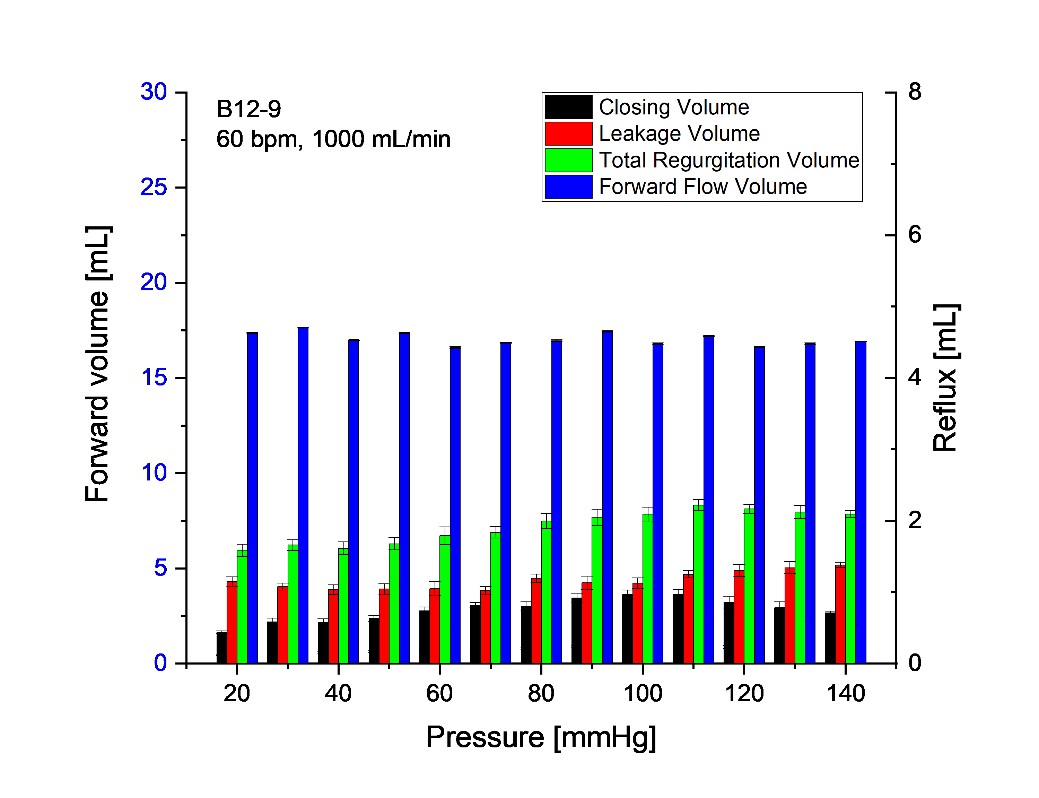
**

**Figure S8: valve B12-9, test series imitating moderate exertion**

**
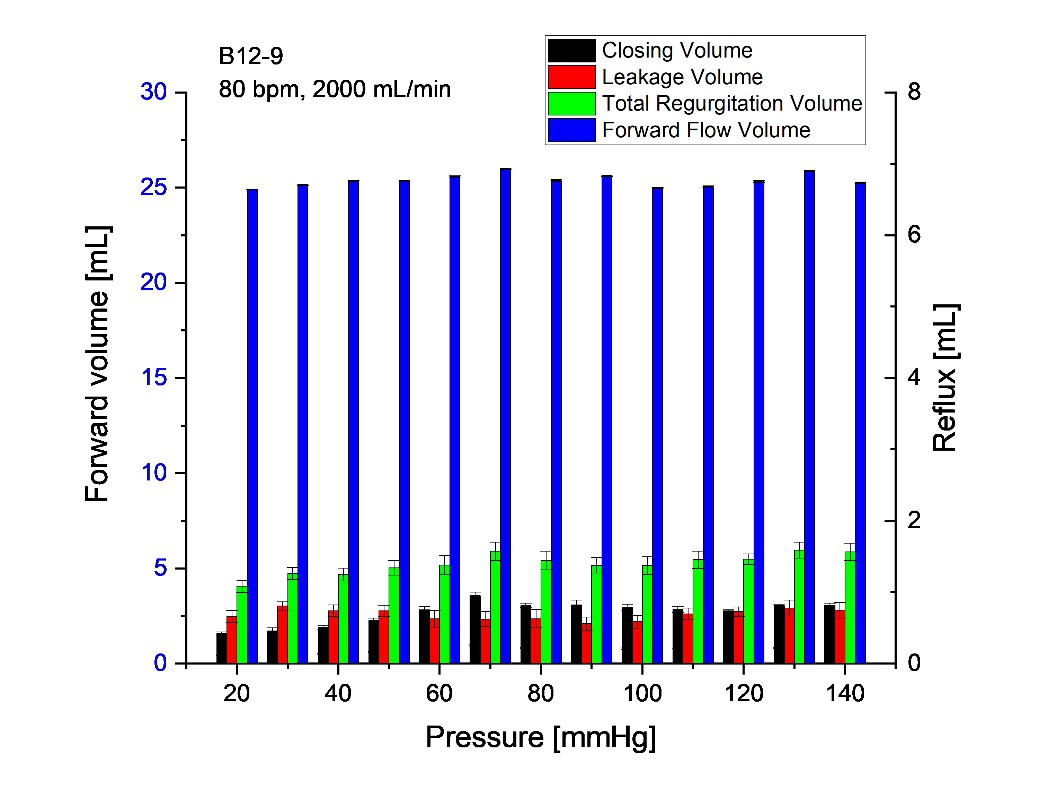
**

**Figure S9: valve B12-9, test series imitating strong exertion**

**
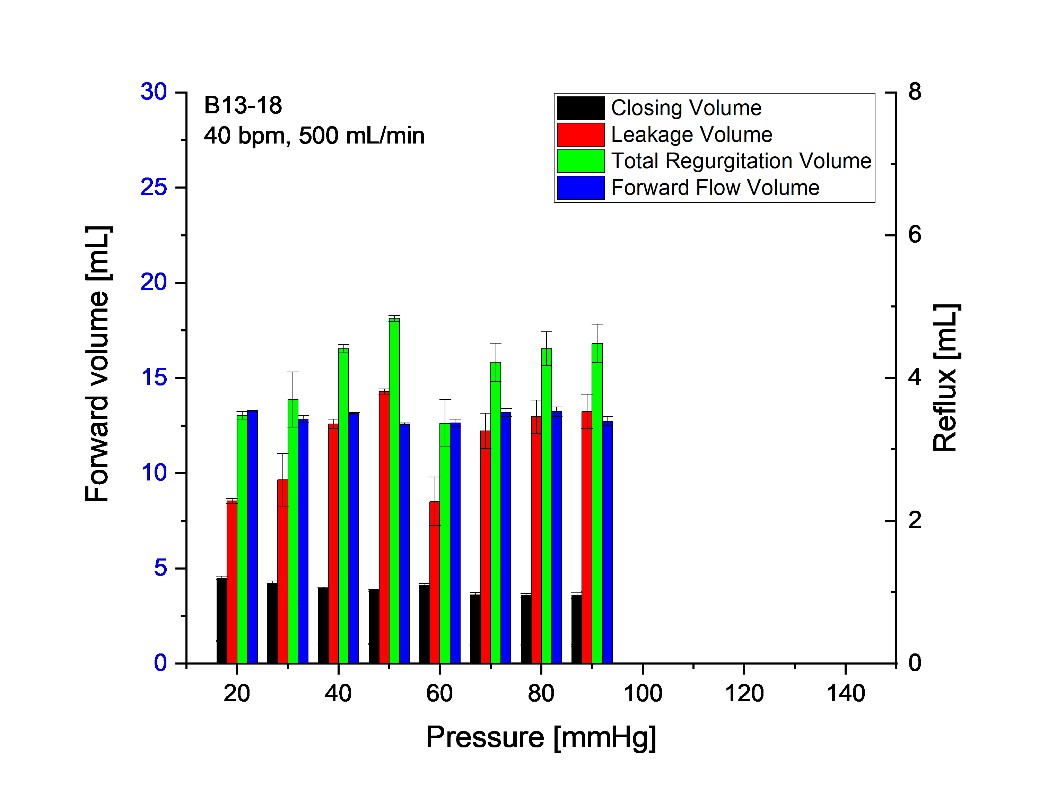
**

**Figure S10: valve B13-18, test series imitating low exertion**

**
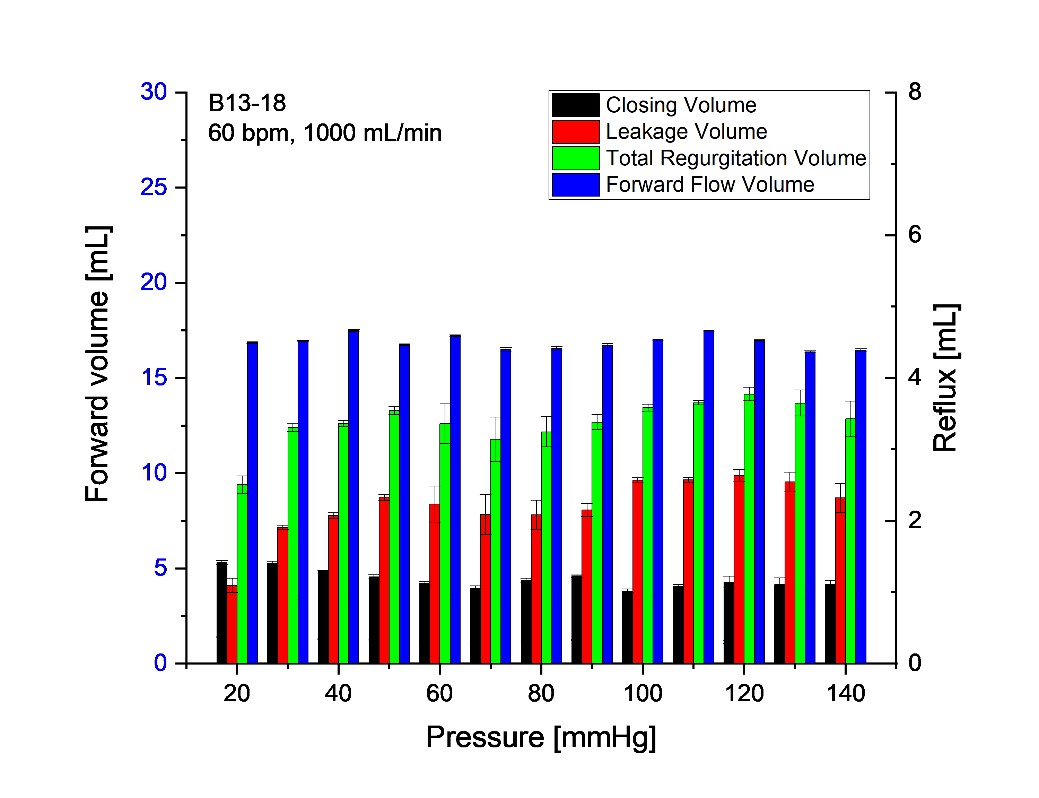
**

**Figure S11: valve B13-18, test series imitating moderate exertion**

**
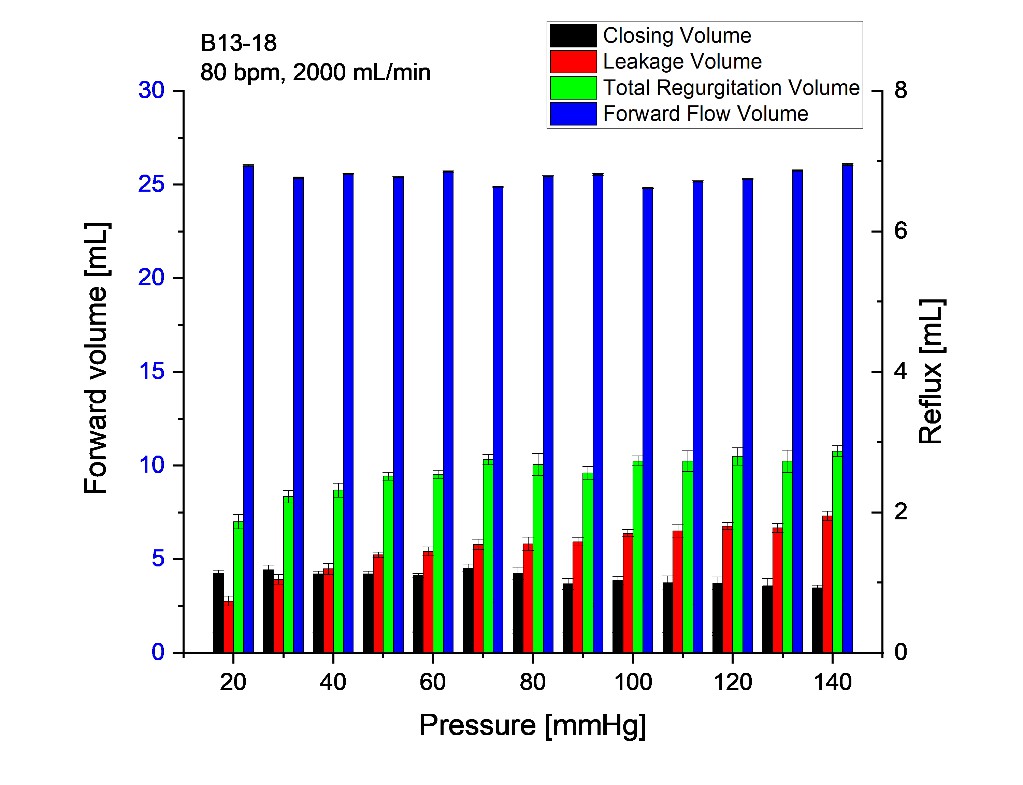
**

**Figure S12: valve B13-18, test series imitating strong exertion**

**
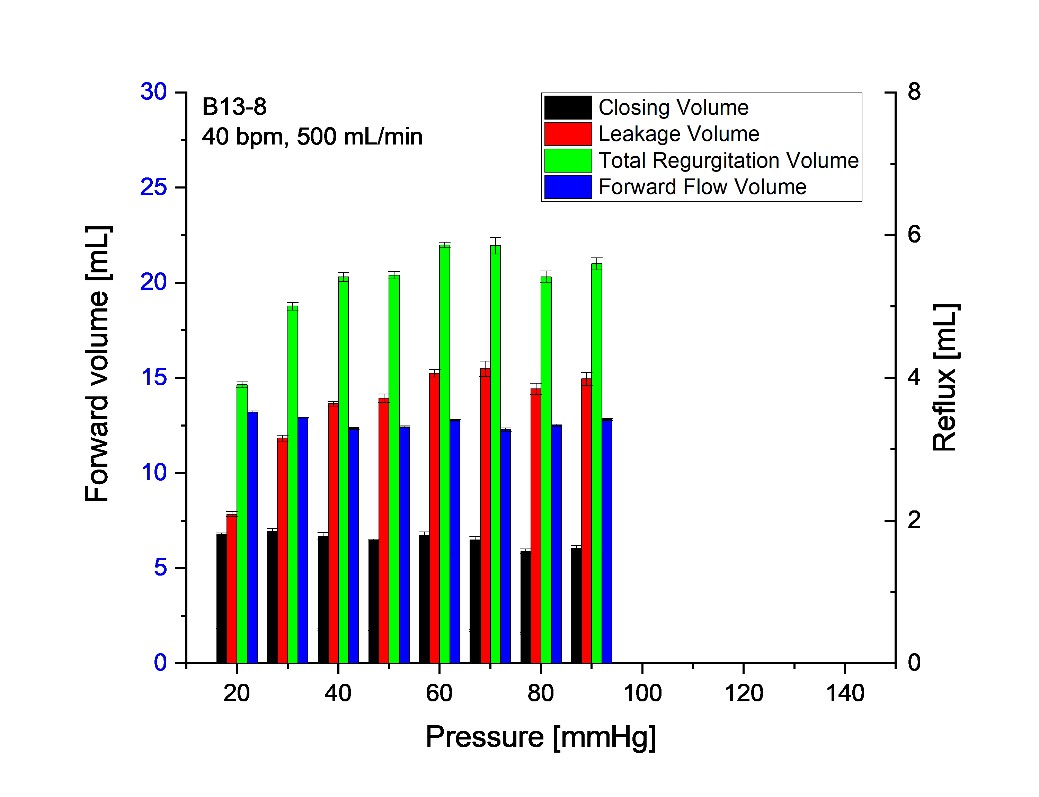
**

**Figure S13: valve B13-8, test series imitating low exertion**

**
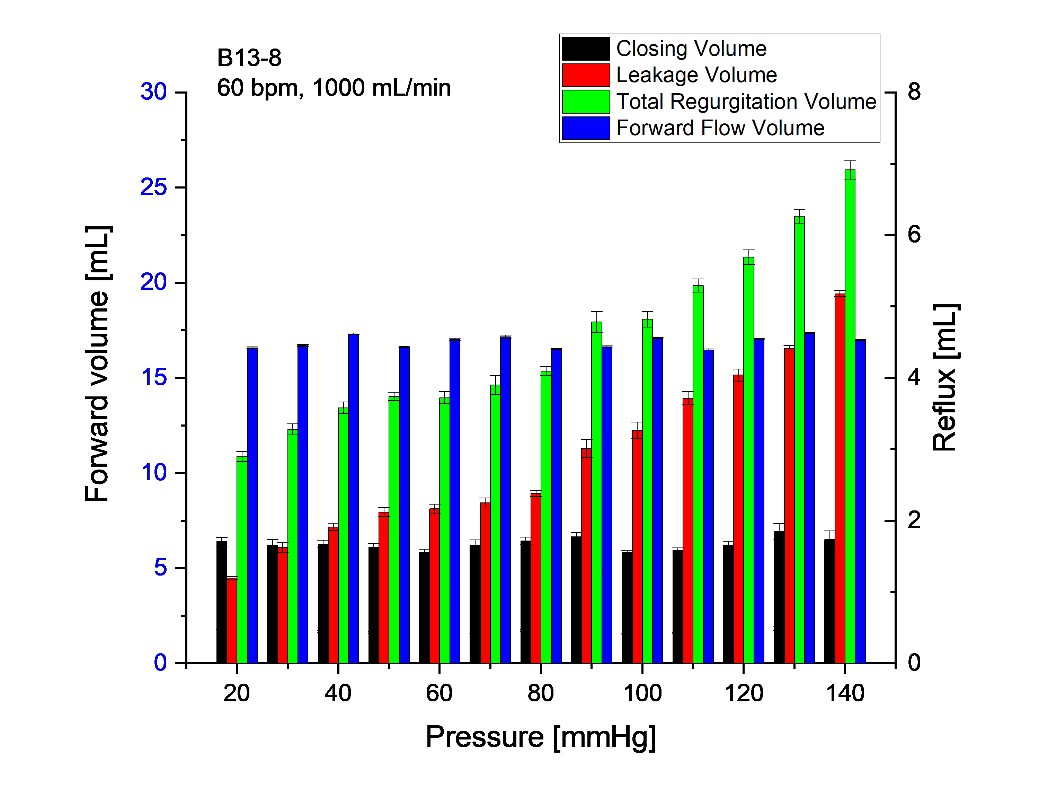
**

**Figure S14: valve B13-8, test series imitating moderate exertion**


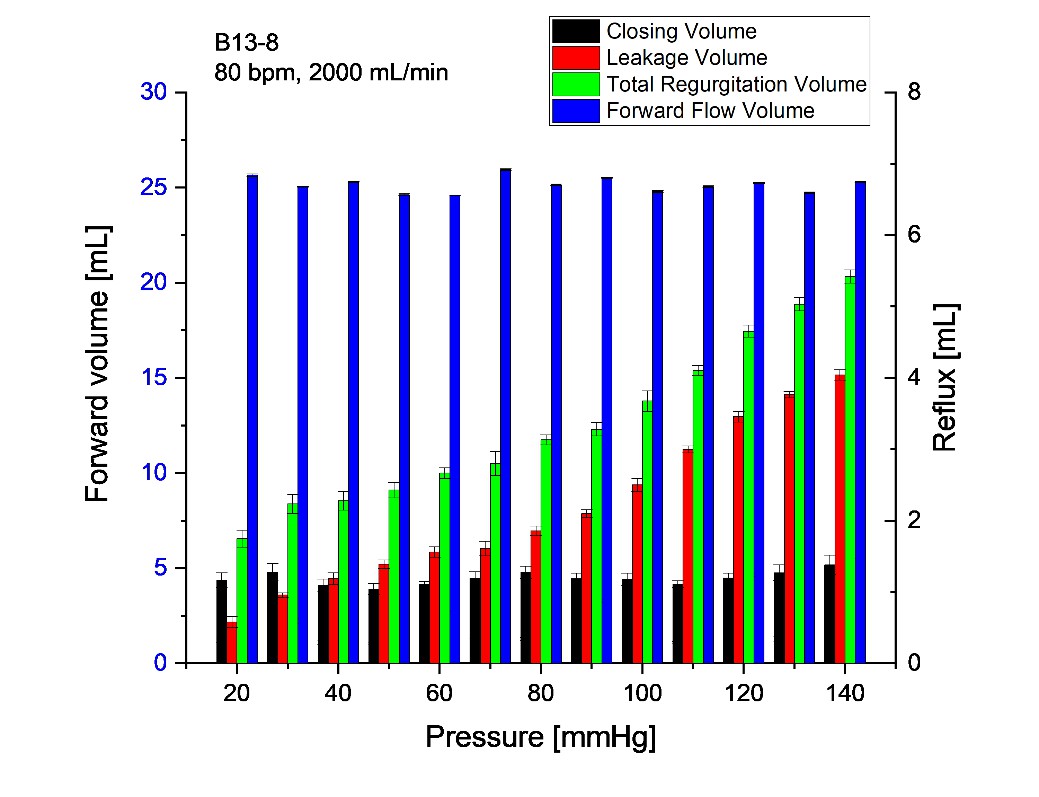


**Figure S15: valve B13-8, test series imitating strong exertion**

The valves were fixed in attachments, which were connected to a water basin (distal pressure forward flow) or a hose (proximal pressure, reflux) for static testing. For pulsatile testing the valve loaded attachments were fixed in the pulse duplicator system, see Figure S 16. As mentioned above, valve A14-17 tilted in static testing at proximal pressure between 60 and 80 mmHg, photographs are displayed in Figure S 16.


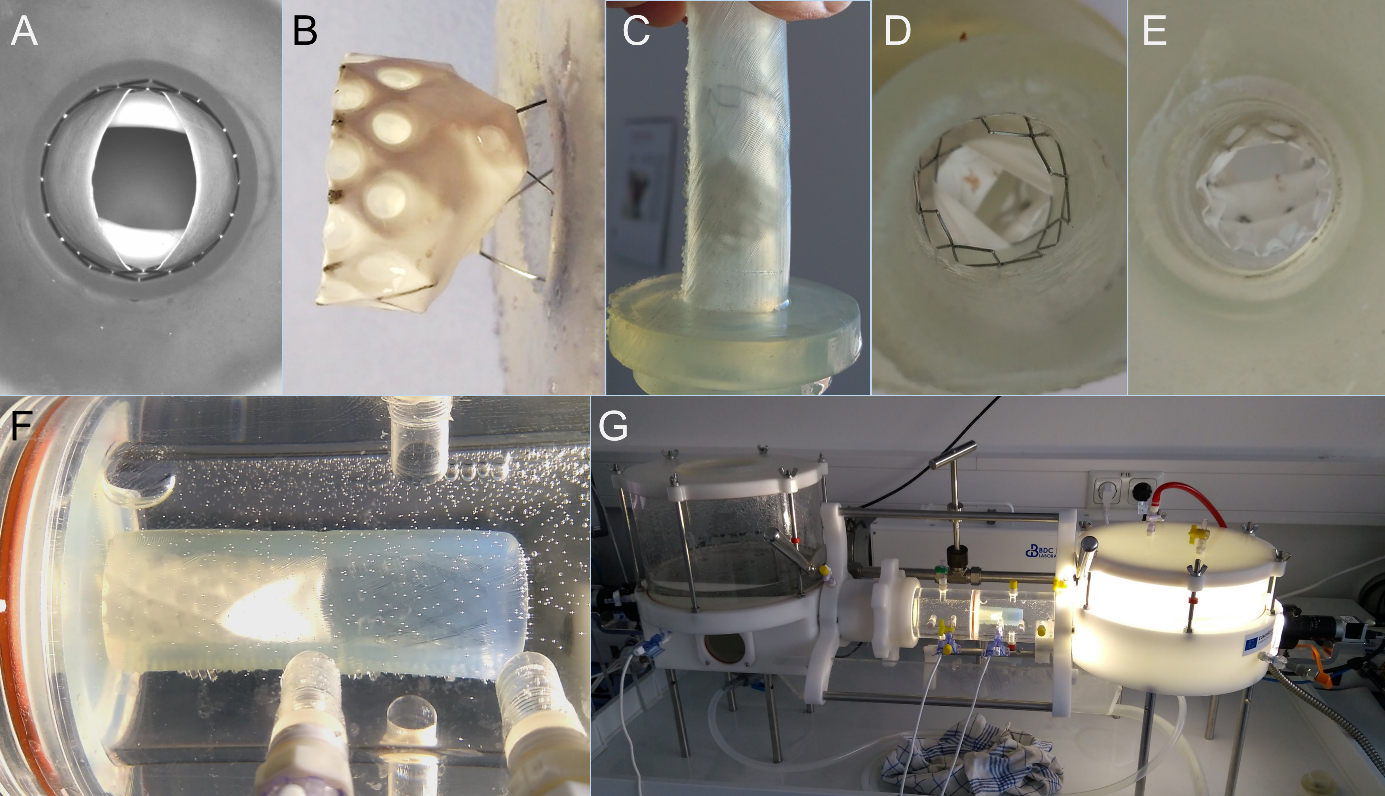


**Figure S 16: Implanted valves.** A: valve A14-17 proximal view on open valve, B: valve A14-17 moved out of the attachment over an additional support ring with leaflets slipped back, C, D, E: valve A14-17 tilted during static testing, in proximal view (D) and distal view (E), F, G: valve B12-9 in pulse duplicator system.

1. **Leaflet deformation**

The leaflet deformation (movement) was visualized according the idea of comparing subsequent images of a video sequence of the inline camera. Areas of the images, which differ from previous image, were highlighted, and unchanged areas were not accentuated (dark). Videos were recoded with 200 frames per second, i.e. a time interval of 5 ms between subsequent images. The video sequences of the inline camera were converted to sequences of single grayscale images of 8 bit depth. For difference image generation each pair of subsequent images were subtracted. The difference images were displayed as heat map using the Look-Up Table “Fire”. Image processing was performed using the software ImageJ, version 1.53n (National Institutes of Health, USA). This method allows the visualization of local leaflet deformation, but no numerical information about the total movement can be derived from these images.

At opening of valve A14-17 the leaflets bulged on both sides evenly and symmetrically, see Figure S17. The valve showed a post-pulse oscillation approximately 0.1 s after closing corresponding to the dark blue flow-curve in Figure 2.


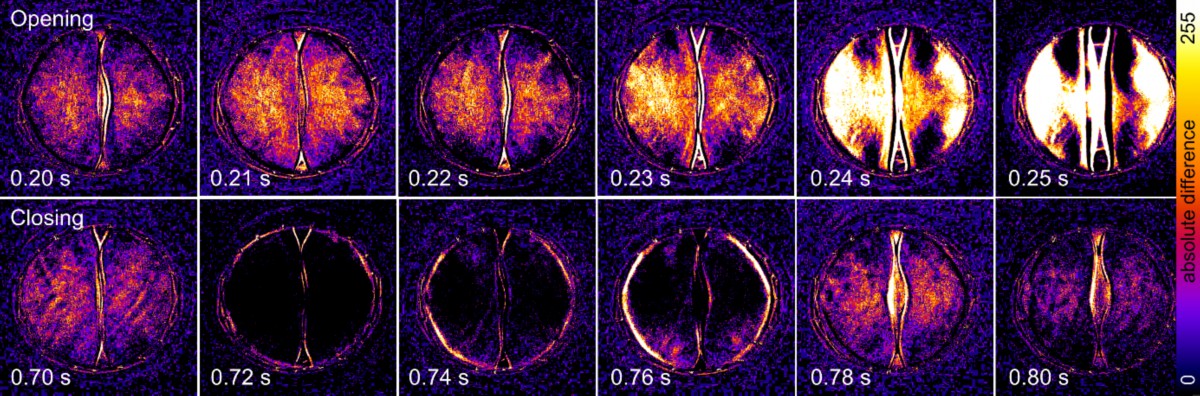


**Figure S17: Visualization of leaflet deformation of valve A14-17.** The difference of pixel values between two subsequent inline camera images of an interval of 5 ms at the indicated time points is displayed as a heat map, no numerical information about the total movement can be derived. At closing a post-pulse oscillation at about 0.8 s appeared.

At opening of valve B12-9 the leaflets bulged symmetrically on both sides and opened simultaneously. A post-pulse oscillation after closing was not observed, see Figure S18.


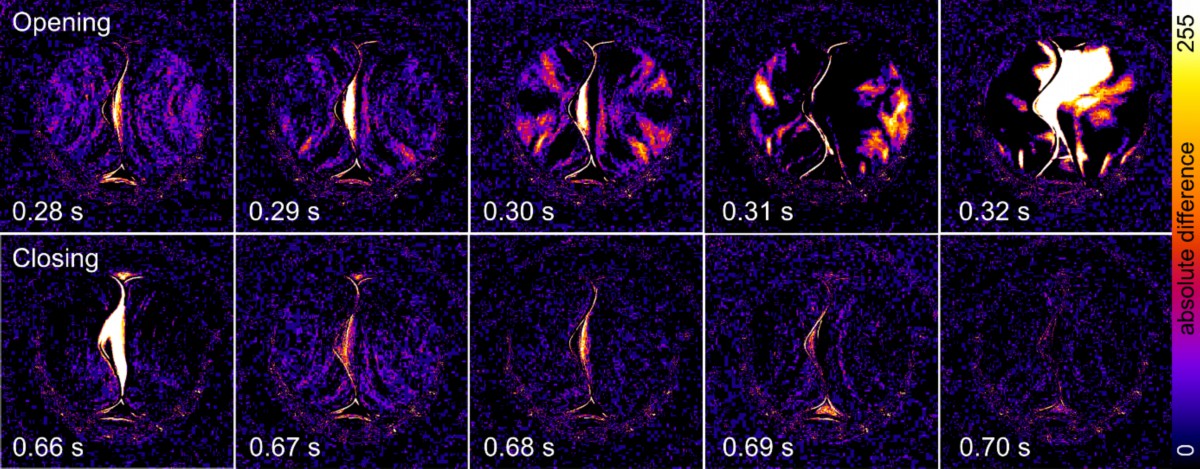


**Figure S18: Visualization of leaflet deformation of valve B12-9.** The difference of pixel values between two subsequent inline camera images of an interval of 5 ms at the indicated time points is displayed as a heat map, no numerical information about the total movement can be derived.

1. **Standards**

There are no standards for in vitro testing of prosthetic venous valves, but standards for heart valves can give orientation, see DIN EN ISO 5840-1 [56].


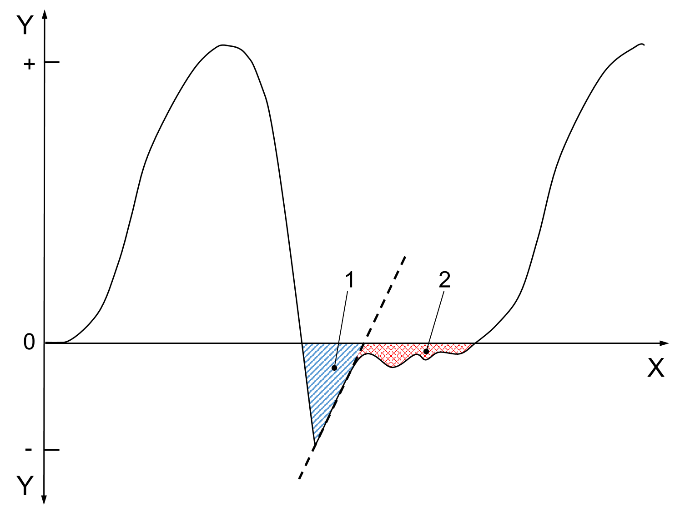


**Figure S19: Test cycle**, X – time, Y – flow, 1 – closing volume, 2 – leakage volume, adapted figure from DIN EN ISO 5840-1 [58]

According to the standard DIN EN ISO 5840-1 [56], the following formula is used for effective orifice area (EOA):


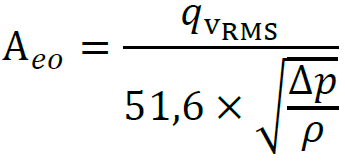


- A𝑒o - effective valve opening area (cm^2^);
- 𝑞v_RMS_ - effective value of the forward flow (mL/s) during the positive differential pressure phase
- Δp the mean pressure difference (measured during the positive differential pressure) (mmHg)
- ρ is the density of the test liquid (g/cm^3^)

with:


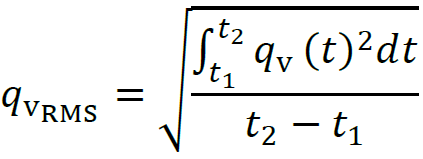


- 𝑞v_RMS_ - effective value of the forward flow during the phase of positive differential pressure
- qv(t) - instantaneous flow rate at time (t)
- t_1_ - time at the beginning of the positive differential pressure phase
- t_2_ - time at the end of the positive differential pressure phase

**Table S2: Selected information from the standard [56].**

| **Standard** | **Table name (translated)** | **Selected information** |
| --- | --- | --- |
| DIN EN ISO 5840-1  (Table 3) | Recommended pressure values for the in vitro test for the left side of the heart - adult population | The peak differential pressure across the closed valve for normal condition is specified with 100 mmHg. |
| DIN EN ISO 5840-2  (Table 1) | Minimum requirements for implant performance, aortal | The maximum total regurgitation fraction (% of forward flow volume) is specified with 10% for small valves (17 mm) up to 20% for large valves (31 mm). |
| DIN EN ISO 5840-3  (Table 1) | Minimum requirements for hydrodynamic in-vitro implant performance, aortal | The maximal regurgitation fraction is specified with 20% for all sizes, including the closing volume, the leak volume across the valve and the paravalvular leak volume. |
